# IgE and IgG4 epitopes of the peanut allergens shift following oral immunotherapy

**DOI:** 10.3389/falgy.2023.1279290

**Published:** 2023-11-29

**Authors:** Ian M. Rambo, Christina M. Kronfel, Adam R. Rivers, Lauren T. Swientoniewski, Jane K. McBride, Hsiaopo Cheng, Reyna J. Simon, Robert Ryan, Stephen A. Tilles, Jacqueline B. Nesbit, Michael D. Kulis, Barry K. Hurlburt, Soheila J. Maleki

**Affiliations:** ^1^ United States Department of Agriculture, Agricultural Research Service, Southern Regional Research Center, New Orleans, LA, United States; ^2^ United States Department of Agriculture, Agricultural Research Service -Genomics and Bioinformatics Research Unit, Gainesville, FL, United States; ^3^ Aimmune Nestlé Health Science, Brisbane, CA, United States; ^4^ Delgado Community College, New Orleans, LA, United States; ^5^ Department of Pediatrics, University of North Carolina School of Medicine, Chapel Hill, NC, United States

**Keywords:** peanut allergy, epitope, immunoglobulin, oral immunotherapy, microarray, ISAC, linear peptide, machine learning

## Abstract

**Background:**

Oral immunotherapy (OIT) with peanut (*Arachis hypogaea*) allergen powder-dnfp (PTAH; Aimmune Therapeutics) is an FDA-approved treatment to desensitize peanut allergic participants.

**Objective:**

Here we assessed shifts in IgE and IgG4 binding to peanut allergens and their epitopes recognized by United States (US) peanut allergic participants (*n* = 20) enrolled in phase 3 PTAH OIT clinical trials.

**Methods:**

Pre- and post- trial participant sera were collected approximately 12 months apart and tested for IgE binding to intact peanut proteins via ImmunoCAP ISAC immunoassays. IgE and IgG4 linear epitopes were identified based on binding to synthetic overlapping 15-mer linear peptides of 10 peanut allergens (Ara h 1-11) synthesized on microarray slides.

**Results:**

Statistically significant decreases in IgE binding were identified for intact Ara h 2, 3, and 6, and known and newly identified IgE epitopes were shown to exhibit shifts towards IgG4 binding post-OIT, with most linear peptides having increased IgG4 binding after treatment with PTAH. While PTAH does not seem to alter the actual peptide binding patterns significantly after one year of treatment, the IgE and IgG4 binding ratios and intensity are altered.

**Conclusion:**

At a population level, the linear IgE and IgG4 epitopes of 10 peanut allergens overlap and that increase in IgG4 with OIT results in displacement of IgE binding to both conformational and linear epitopes. Furthermore, it appears as though the increase in IgG4 is more important to achieve desensitization at the 12-month timepoint than the decrease in IgE. This type of knowledge can be useful in the identification of IgE and IgG4-binding allergen and peptide biomarkers that may indicate desensitization or sustained unresponsiveness of allergic individuals to peanut.

## GRAPHICAL ABSTRACT

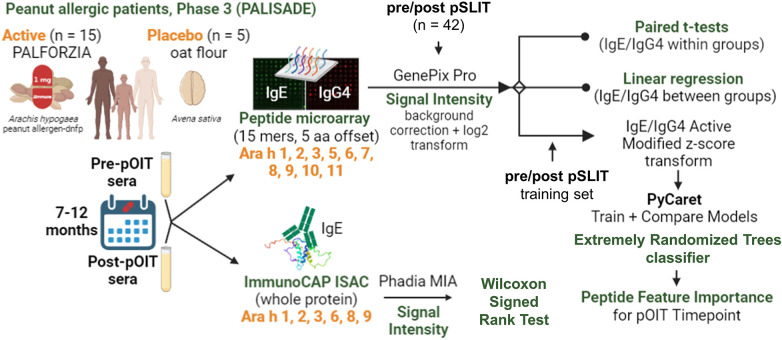
 Overview of methods used in the analysis of samples from the PALISADE study.

## 1. Introduction

Peanut allergy is an immunologic disease characterized by the production of peanut-protein-targeting immunoglobulin E (IgE) antibodies that mediate reactions ranging from mild cutaneous manifestations to life-threatening systemic anaphylaxis. The risk of life-threatening allergic reactions has substantial negative impacts on quality of life for nearly 10% of adults (1) and 8% of children worldwide that live with food allergies (2), while costing an estimated $25 billion per year in the United States alone (1, 3). Peanut oral immunotherapy (pOIT) is a promising treatment to desensitize participants by increasing tolerance via administration of small, escalating doses of allergen over several months (4, 5) and is shown to be safe and highly successful in suppressing IgE-mediated allergic responses, i.e., desensitization (6). However, pOIT participants can experience gastrointestinal issues and allergic reactions (7), and participants who do not continue ingestion of the allergenic food after pOIT exhibit a high rate of regression to an allergic state (7, 8). Repeated oral administration of allergen during pOIT is shown to decrease peanut-specific IgE and increase peanut-specific IgG4, both of which may play roles in desensitization (9–14). Determining the modulation of IgE and IgG4 epitopes during desensitization might inform safer pOIT regimens, as pOIT has been shown to significantly increase peanut-specific IgG4 based on direct serum antibody measurements and linear peptide binding (13, 15). Here, we utilize linear peptide microarrays and intact allergen ImmunoCAP Immuno Solid-phase Allergen Chip (ISAC) immunoassays to determine IgE and IgG4 epitope shifts over the course of the PTAH PALISADE pOIT phase 3 clinical trial.

## 2. Materials and methods

### 2.1. Human sera

Sera were collected from peanut-allergic participants (*n* = 20) during the Aimmune Therapeutics funded peanut allergy oral immunology clinical studies, ARC001 (16) (NCT01987817) and PALISADE (17) (ARC003, NCT02635776) after informed consent. Participant allergy history was confirmed by clinical history and double-blind placebo controlled oral food challenges (DBPCFC). Phase 3 trials for PTAH (peanut allergen dnfp, PALFORZIA®) were administered with DBPCFC, with the Active group (*n* = 15) receiving PTAH and the Placebo group (*n* = 5) receiving oat flour (*Avena sativa*). Sera were collected prior to pOIT trials and after 12 months of treatment. Participants were removed from trials if they had anaphylactic reactions to peanut. Baseline and exit maximum tolerated dose (MTD) of peanut were administered to participants before and after pOIT and ranged from 3 to 1,000 mg, and all participants undergoing the trial passed this oral food challenge. Baseline and exit skin prick tests (SPTs) were administered to gauge IgE reactivity via mean wheal diameter (MWD) measurements at pre and post timepoints (Table 1).

**Table 1:** Participant information pre and post pOIT.

#	TG	Age	S	BL tIgE IU/ml	Exit tIgE IU/ml	BL psIgE kUA/l	Exit psIgE kUA/l	BL psIgG4 mgA/l	Exit psIgG4 mgA/l	BL MWD	Exit MWD	BL MTD (mg)	Exit MTD (mg)	Other allergy	psIgE RC	psIgG4 RC
003-012	A	8	M	458	1,332	41.10	68.30	.09	8.11	13.0	12.5	30.0	1,000	FA, AR	66.18005	8,911.11
004-007	A	12	F	619		170.50		.41		12.0		10.0		As, AD, AR		
006-011	A	15	M	882	995	442.00	544.0	.32	29.90	9.0	6.5	10.0	600	As, FA, AR	23.08	9,243.75
006-013	A	14	F	308	519	77.80	251.0	.70	9.78	10.0	17.0	10.0	1,000	As, AD, AR	222.62	1,297.14
018-001	A	23	M	1,572	1,204	5.54	5.43	.45	.69	13.5	11.0	10.0	600	As, FA, AR	−1.98	53.33
018-005	A	32	F	201	253	19.50	69.40	.65	9.53	12.0	9.0	10.0	1,000	A, RA	255.90	1,366.15
031-006	A	13	F	1,022	676	289.00	247.5	.32	2.88	11.5	9.0	30.0	1,000	As, FA, AR	−14.36	800
031-010	A	13	F	155	170	31.50	42.50	1.31	15.70	7.5	5.5	10.0	1,000	As, AR, FA	34.92	1,098.47
032-010	A	12	M	835		304.50		2.20		9.0	7.5	3.0	1,000	As, AD, FA		
032-012	A	13	M	406		274.00		.30		7.5	9.0	30.0	1,000	FA, RA		
035-013	A	34	F	119	135	.79	2.91	.38	.57	11.0	10.0	10.0	600	As, AD, FA, AR	268.35	50.00
062-004	A	24	M	91	148	32.40	42.20	1.73	8.66	10.0	9.5	30.0	1,000	FA	30.25	400.58
062-009	A	10	F	2,781	1,491	61.10	52.70	.48	2.23	12.5	7.5	3.0	300	As, FA, AR	−13.75	364.58
062-014	A	14	M	784	668	4.33	2.50	.18	.24	13.5	7.0	30.0	1,000	As, DA, FA	−42.26	33.33
062-017	A	13	M	59		17.60		.26		18.0	14.5	30.0	100	As, AD, FA		
032-009	P	15	M	344		98.70		.66		13.5	16.0	3.0	100	As, AD, FA		
032-014	P	10	M	999		3.68		1.73		14.5	10.5	10.0	3	As, AD, FA		
032-015	P	15	F	1,072	1,021	6.00	3.56	.19	.10	12.5	18.0	30.0	30	As, AR	−40.67	−47.37
062-005	P	17	M	4,268	2,354	100.00	1,358.00	5.87	1.90	7.0	10.0	30.0	30	As, AD, AR	1,258.00	−67.63
062-008	P	17	M	4,268	2,354	100	1,358.00	5.87	1.90	7.0	10.0	30.0	30	As, AD, AR	1,258.00	−67.63

ID, participant ID; A, active; P, placebo; TG, treatment group; Age, age at enrollment; S, sex, BL, baseline; tIgE, total IgE; psIgE, peanut specific IgE; MWD, mean wheel diameter; cm, centimeter, mg, milligram MTD, maximum tolerate dose; OA, other allergy; As, asthma; FA, food allergy; AD, atopic dermatitis; AR, allergic rhinitis; RC, relative change. Blank cells indicate certain measurements that were not taken for a participant at the study exit.

### 2.2. Peptide microarrays

Microarrays were utilized for detection of participant sera IgE and IgG4 binding to linear peanut peptides. Synthetic 15-mer linear peptides offset by 5 amino acids that represented the entire amino acid sequences of Ara h 1, 2, 3, 5, 6, 7, 8, 9, 10, and 11 (Supplementary Table 1) were commercially synthesized and spotted in triplicate onto microarray slides by JPT Peptide Technologies (Berlin, Germany). Slide preparation was performed as previously described (18). Slide hybridization was performed on an HS400 Pro Hybridization Station (Tecan, San Jose, CA, USA), with each slide placed in an individual chamber. Slides were blocked in 200 μl filtered SuperBlock (Thermo-Fisher, Waltham, MA, USA) for 30 min at room temperature (RT) under agitation, then washed for 2 min with Tris-buffered saline containing polysorbate-20 (100 mM Tris, 274 mM NaCl, 5.4 mM KCl, and 0.5% Tween-20, TBST). Participant sera were injected into slide chambers and incubated at 4°C overnight (∼16 h) with agitation. Slides were washed as described above before injecting with mouse anti-human IgE (Life Technologies, Grand Island, NY) diluted in SuperBlock (3.3 μg/ml), incubated for 30 min at RT, washed and incubated with diluted Cy3-conjugated goat anti-mouse IgG (0.4 μg/ml; Life Technologies) for 30 min at RT. The wash step was repeated, and slides were dried with N_2_ gas. Washed and dried slides were scanned with a GenePix-4000B scanner (Software: GenePix Pro 7; Molecular Devices, San Jose, CA, USA) to detect IgE binding measured by Cy3-green (532 nm). Slides were then washed and incubated with 160 ul of anti-human IgG4 (rabbit), incubated for 30 min at RT, washed, and incubated with anti-rabbit Alexaflour-red for 30 min. Slides were scanned for IgG4 signals at 635 nm with a GenePix-4000B scanner.

### 2.3. Microarray data processing, normalization and epitope identification

GenePix GPR output files containing IgE and IgG4 signal intensities were processed with the Limma package v3.52.4 using R v4.2.1 and RStudio v2022.07.0. Median feature pixel intensities at 532 nm (IgE) and 635 nm (IgG4) were background corrected using the backgroundCorrect.matrix function in Limma (offset = 10, method = “normexp”) and were base 2 logarithm transformed, and the median for each background-corrected peptide triplicate was taken. Cyclic-Loess normalization for each peptide was performed between all possible pairs of arrays for 3 iterations, with a loess smoothing window span of 0.3 using the normalizeBetweenArrays function (method = “cyclicloess”, cyclic.method = “pairs”).

Median peptide fluorescence signal-to-noise ratios (SNRs) across microarray chip lots were used to determine major reactive IgE and IgG4 epitopes. Positive IgE or IgG4 binding was identified as a median SNR >3, which is based on previous chicken sera controls having SNR values of 3. Peptides with positive IgE or IgG4 binding by at least 50% of the sera (i.e., 50% of participant sera had median SNRs >3) were considered major epitopes (19). Peptides were mapped to 3D protein structures using the Protein Data Bank 3D viewer (20).

### 2.4. Within and between-group comparisons of pre- and post-pOIT peptide intensities

To identify shifts in peptide binding intensity from pre- to post-pOIT within the Active and Placebo treatment groups, paired *t*-tests of IgE/IgG4 binding intensity ratios (IgE/IgG4) were performed in R v4.2.1 [*t*-test(paired = TRUE), stats package v4.2.1] for each 15-mer linear peptide from pre- to post-pOIT. Prior to conducting the paired *t*-tests, the approximate normality of peptide binding intensity distributions were assessed with quantile-quantile plots (Supplementary Figures S1–S10) generated with the geom_qq() and geom_qq_line() functions in R with ggplot v3.4.2. False discovery rate control of paired *t*-test *p* values was performed via Benjamini-Hochberg (FDR) correction (21), adjusting for multiple comparisons with a protein (i.e., peptide-level *t*-tests were penalized based on the number of total peptides within their respective protein) and imposing an adjusted *p* value threshold <0.05. Peptides with significant adjusted *p* values were considered to have undergone a significant IgE/IgG4 ratio increase or decrease over the course of the study if their mean difference was >1 or <1, respectively. Cohen's *d* effect size for paired samples (22, 23) was calculated for each peptide (pre vs. post within treatment groups) and corrected to the Hedges’ *g* for paired samples (24).

Between-group (i.e., Active vs. Placebo) comparisons of per-peptide IgE/IgG4 were performed with linear regression models (LM) using the lm() function in R: *IgE/IgG4 Post = IgE/IgG4 Pre + Treatment*. LM fit metrics are available in Supplementary Table 2. Raw *p* values were adjusted for multiple testing via FDR correction (21). Contrasting least-squares means were computed using the emmeans v1.8.7 package (25). Baseline-adjusted geometric mean post-OIT IgE/IgG4 values were compared per peptide for Active and Placebo groups to determine whether the Active group finished the study with significantly lower or higher IgE/IgG4 ratio compared to Placebo for that peptide, assuming both groups began the study with the same antibody binding intensity. Known epitopes for Ara h 1, 2 and 3 were obtained from the Structural Database of Allergenic Proteins (SDAP) v2.0 (26). Linear B-cell epitopes for Ara h 5-11 were predicted with the BepiPred 2.0 webserver (27), and predicted epitopes with scores >5 were considered in downstream analysis.

### 2.5. Correlation networks

Correlation matrices were constructed from normalized log2 IgE and IgG4 intensities for pre- and post-pOIT, with pairwise Pearson correlation performed between pre- and post-pOIT matrices via the correlate() function in the corrr R package v0.4.4 (28) (parameters: use = “pairwise.complete.obs”, method = “pearson”). Correlation networks were visualized using the visNetwork v2.1.2 package in R.

### 2.6. Supervised binary classification

Supervised binary classification models were used to predict desensitization to peanuts and determine which peanut allergen peptide features were most important for those models. IgE/IgG4 of peanut-allergic participant (*n* = 42) sera from a separate peanut sublingual oral immunotherapy (pSLIT) study utilizing identical microarray-bound peptides and a pre/post experimental design with comparable treatment durations (29) were used for train and test sets (80% train, 20% test), and Active group PALISADE phase 2 (*n* = 2) and phase 3 (*n* = 15) participant pOIT IgE/IgG4 were used as the validation (unseen) data set. The pSLIT and PALISADE GPR outputs were processed together with the Limma package v3.52.4 using R v4.2.1, as described in Methods section 2.3. Modified *z*-scores of per-peptide IgE/IgG4 (features) and pre/post timepoints (labels) were used for supervised binary classifiers using the Python machine learning libraries PyCaret v2.3.10 (30) and SciKit-Learn v0.23.2 (31). Feature selection was based on the importance of weights (Scikit-learn SelectFromModel using LightGBM estimator) using mean importance value thresholds of 0.6, 0.7, and 0.8. For each importance value threshold, 40 sessions comparing classifier performance were run in PyCaret using different random seeds states ranging from 0 to 1,000. Within each session (*n* = 120), train and test sets were sampled from the pSLIT dataset (80% train, 20% test), and all base classification estimators available in the SciKit-Learn v0.23.2 library were trained and evaluated with Stratified KFold cross validation (splits = 10) on the training set. The top 4 models were selected based on accuracy, and hyperparameter tuning was performed for these models with a Random Grid Search with 50 iterations using Stratified KFold cross validation (splits = 10) with F1 score as the optimization metric. The original models were used in downstream analysis if they outperformed their tuned versions. Ensemble methods were performed via bagging (32), stacking (33), and boosting (34), using 15 base estimators, with F1 score as the optimization metric. For all the generated models, the base estimator or ensemble estimator with the highest F1 score was returned, finalized with the entire train and test datasets, and predictions were performed on the unseen PALISADE dataset. The predictive performance on the unseen dataset for each finalized model was compared and the best model was selected based on F1 score and Matthews correlation coefficient (MCC) (35). A tree-based ensemble classifier [Extremely Randomized Trees (ERT) classifier (36)] was selected [parameters: class_weight='balanced', criterion = 'entropy', max_depth = 2, max_features = 'sqrt', min_impurity_decrease = 0.0005, min_samples_leaf = 5, min_samples_split = 7, n_estimators = 200, n_jobs = −1(36)] was selected as the best performing model (FST = 0.6, random_state = 388). Gini Impurity (37) was used to identify peptide features with the largest effect on this particular ERT model's prediction of desensitization.

### 2.7. ImmunoCAP ISAC immunoassays

ImmunoCAP Immuno Solid-phase Allergen Chip (ISAC) immunoassays (Thermo-Fisher, Upsala, Sweden) were used to measure pre- and post-pOIT participant sera IgE binding to purified intact Ara h 1, 2, 3, 6, 8, 9 proteins. IgE signal intensity levels in ISAC Standardized Units (ISU-E) with an operating range of 0.3–100 ISU-E were generated by Phadia Microarray Image Analysis software v1.2 (Thermo-Fisher Scientific, Waltham, MA, USA). IgE intensities were log2 transformed [log2(x + 1)]. Wilcoxon signed rank tests were used to compare IgE binding intensity distributions from pre to post for each protein using the wilcox.test() function in the R stats package v.4.2.1 (parameters: paired = TRUE, alternative = “two.sided”, exact = TRUE, conf.level = 0.95). Spearman correlation matrices were constructed using the cor() function in the R stats package v4.2.1, and correlation plots were made using the corrplot v0.92 package in R.

## 3. Results

### 3.1. IgE binding to intact allergens

**Figure 1:**
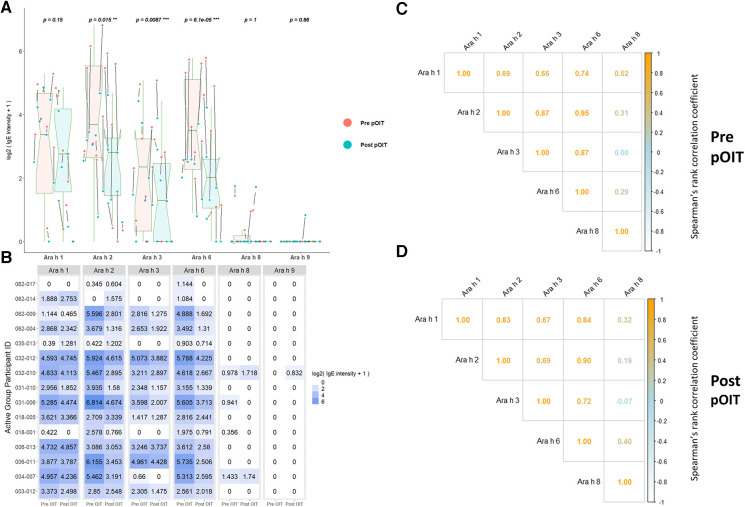
ImmunoCAP ISAC ­­immunoassays indicate significantly reduced IgE binding to intact Ara h 2, 3, and 6 post pOIT for active group participants. (**A**) ISAC Pre vs. post pOIT for participant population. *P* values are for paired Wilcoxon signed rank tests, with ** indicating *p* < 0.05 and *** for *p* < 0.01. Arrows between dots indicate pre-to-post log2 IgE intensity shifts for individual participants. Box plot notches indicate the 95% confidence interval of the median. (**B**) Log2 transformed IgE intensity values per protein for Active group participants, pre and post pOIT. (**C**) Spearman correlations of IgE intensity values for Active group participants, pre pOIT. (**D**) Spearman correlations of IgE intensity values for Active group participants, post pOIT.

ISAC immunoassays were utilized for IgE binding to whole proteins to determine conformational aspects to binding. PALISADE participant IgE binding was shown via Wilcoxon signed rank tests to have significantly different distributions from pre-to-post pOIT for Ara h 2 (*p* = 0.018), Ara h 3 (*p* = 0.0087), and Ara h 6 (*p* = 0.00073), with IgE binding exhibiting an overall decrease in the study population after pOIT (Figure 1A). The Ara h 1 IgE binding distributions of the Active participant group were not significantly different from pre to post pOIT (*p* = 0.15). The total IgE binding intensity to Ara h 8 and Ara h 9 was much lower compared to Ara h 1, 2, 3, and 6. Ara h 8 binding increased for two and decreased for two participants, however all other participants did not exhibit notable IgE binding shifts to this protein and pre-to-post distributions were not significant (*p* = 1) (Figures 1A,B). Similarly, only one participant showed increased binding to Ara h 9 from pre-to-post, while all other participants had no notable binding and pre-to-post distributions were not significantly different (*p* = 0.86; Figures 1A,B). Ara h 8 IgE binding was not strongly correlated with any other intact protein at pre and post-pOIT timepoints (Figures 1C,D). On a per-patient basis, those with higher IgE binding to Ara h 2 exhibit higher binding to the homologous 2S albumin Ara h 6 (Figure 1B), with a population-level Spearman correlation coefficient of 0.95 at the pre-pOIT timepoint (Figure 1C) and 0.9 at post-pOIT (Figure 1D).

### 3.2. IgE and IgG4 binding to linear peanut allergen peptides

**Figure 2:**
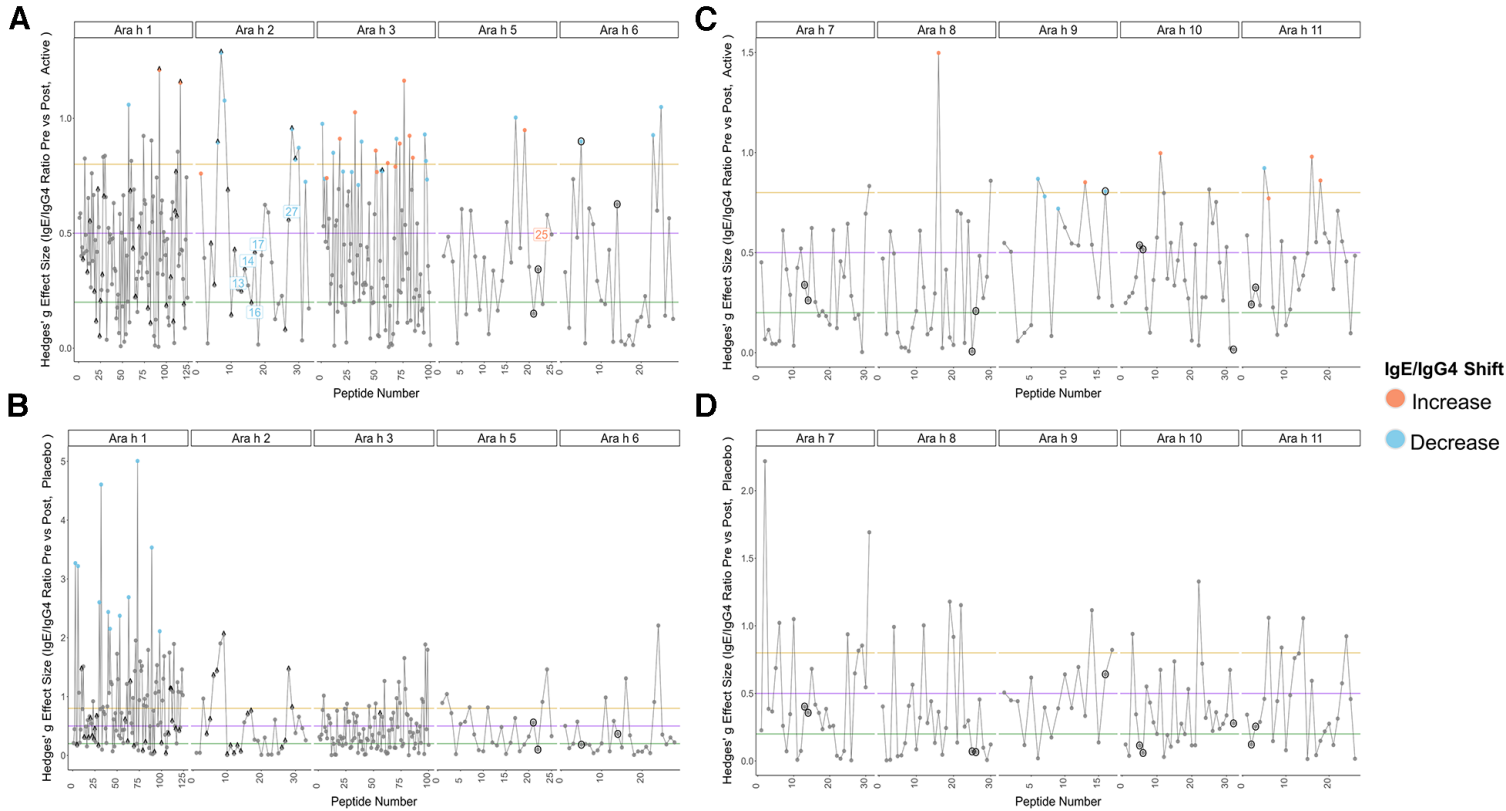
Pre-to-post pOIT IgE/IgG4 indicate increased shifts from IgE to IgG4 binding in active vs. Placebo group. (**A**) Active group, Ara h 1, 2, 3, 5, 6. (**B**) Placebo group, Ara h 1, 2, 3, 5, 6. (**C**) Active group, Ara h 7, 8, 9, 10, 11. (**D**) Placebo group, Ara h 7, 8, 9, 10, 11. Orange and blue dots respectively indicate peptides with a significant increase or decrease in IgE/IgG4 ratio from pre-to-post pOIT based on within-group paired *t*-tests, as determined by an FDR-adjusted *p* value <0.05. Blue and orange number labels respectively indicate peptides with a significant decrease or increase in pre-to-post pOIT IgE/IgG4 ratio for Active compared to Placebo based on FDR-adjusted *p* values <0.15 for an LM. Horizontal lines at y = 0.2, 0.5, and 0.8 respectively indicate boundaries for small, medium, and large effect size benchmarks (22). Points with “^” indicate peptide sequences with regions matching previously identified IgE epitopes listed in the SDAP database, while black circles around points indicate linear peptides predicted with BepiPred v2.0.

Pre to post-pOIT linear epitope shifts were identified using IgE/IgG4. In general, significant shifts in antibody binding occurred within regions of overlapping peptides. Based on paired *t*-tests, Ara h 1 had 1.6% of peptides exhibiting significantly increased IgE/IgG4 (increasing IgE and/or decreasing IgG4) from pre- to post-pOIT in the Active group, while 0.8% of peptides had significant shifts towards decreased IgE/IgG4 (indicating increasing IgG4 and/or decreasing IgE binding) (Figure 2A, Table 2). Known epitope regions containing significantly different peptides include those containing peptides 5–7 (IgE/IgG4 decrease), peptides 15–22 (IgE/IgG4 decrease), peptides 28–31 (IgE/IgG4 decrease), and peptide 116 (IgE/IgG4 increase). In the Placebo group, 8.87% of Ara h 1 peptides underwent a significant IgE/IgG4 decrease (Table 2, Figure 2B).

**Table 2:** Percentage of peptides that had significant IgE/IgG4 ratio shifts from pre- to post-pOIT based on study population paired *t*-tests.

Protein	Ratio shift	% Peptides with significant shift (paired *t*-test)	Treatment group
Ara h 1	IgE/IgG4 Decrease	0.806	Active
Ara h 1	IgE/IgG4 Increase	1.613	Active
Ara h 1	NS	97.581	Active
Ara h 2	IgE/IgG4 Decrease	21.212	Active
Ara h 2	IgE/IgG4 Increase	3.03	Active
Ara h 2	NS	75.758	Active
Ara h 3	IgE/IgG4 Decrease	13	Active
Ara h 3	IgE/IgG4 Increase	11	Active
Ara h 3	NS	76	Active
Ara h 5	IgE/IgG4 Decrease	4	Active
Ara h 5	IgE/IgG4 Increase	4	Active
Ara h 5	NS	92	Active
Ara h 6	IgE/IgG4 Decrease	10.714	Active
Ara h 6	NS	89.286	Active
Ara h 7	NS	100	Active
Ara h 8	IgE/IgG4 Increase	3.333	Active
Ara h 8	NS	96.667	Active
Ara h 9	IgE/IgG4 Decrease	23.529	Active
Ara h 9	IgE/IgG4 Increase	5.882	Active
Ara h 9	NS	70.588	Active
Ara h 10	IgE/IgG4 Increase	3.125	Active
Ara h 10	NS	96.875	Active
Ara h 11	IgE/IgG4 Decrease	3.846	Active
Ara h 11	IgE/IgG4 Increase	11.538	Active
Ara h 11	NS	84.615	Active
Ara h 1	IgE/IgG4 Decrease	8.871	Placebo
Ara h 1	NS	91.129	Placebo
Ara h 2	NS	100	Placebo
Ara h 3	NS	100	Placebo
Ara h 5	NS	100	Placebo
Ara h 6	NS	100	Placebo
Ara h 7	NS	100	Placebo
Ara h 8	NS	100	Placebo
Ara h 9	NS	100	Placebo
Ara h 10	NS	100	Placebo
Ara h 11	NS	100	Placebo

IgE/IgG4 increases, increased IgE and/or decreased IgG4 binding; IgE/IgG4 decreases, decreased IgE and/or increased IgG4 binding; NS, peptides that did not have statistically significant IgE/IgG4 ratio shifts.

For the major peanut allergen Ara h 2 (Ara h 2.0201), 21.2% of the 15-mer peptides exhibited increased IgE/IgG4% and 3% exhibited decreased IgE/IgG4 over the 12-month pOIT period within the Active group based on paired *t*-tests, while no significant changes were observed in the Placebo group (Table 2). Peptide regions 6–8 and 28–30 form known IgE epitopes comprising *α*-helices that span protein segments I, III, and IV (38) (39), and these displayed decreased IgE/IgG4 within the Active group based on paired *t*-tests (Figure 2A). IgE/IgG4 decreased for peptide 31 in the Active group over the course of pOIT with moderate effect size (Figure 2A) and may be a potential IgE and IgG4 epitope based on median SNRs from part of the population (Supplementary Figure 1^2^). This may indicate a previously undescribed linear epitope in segment IV of Ara h 2 that has undergone a shift from IgE to IgG4 binding in the Active group over the course of pOIT. Linear regression analysis did not identify any peptides with significant within-group IgE/IgG4 shifts where the Active group finished the study with significantly higher or lower IgE/IgG4 vs. Placebo group. Using an adjusted *p* value cutoff <0.05, only Ara h 2 peptide 17 was identified as being significantly different (Active group finished with significantly lower IgE/IgG4). Using a more liberal adjusted *p* value cutoff of <0.15 to adjust for small library size, known IgE epitope regions (38) spanning Ara h 2 peptides 13 (*p *= 0.1), 14 (*p = *0.1), 16 (*p *= 0.059), 17 (*p* = 0.046), and 27 (p = 0.1) were shown to have lower IgE/IgG4 in the Active group vs. Placebo group at the end of pOIT.

The major peanut allergen Ara h 3 (11S storage protein Ara h 3.0101) had 11% of peptides undergo IgE/IgG4 increases and 13% undergo IgE/IgG4 decreases in the Active group, with no significant changes in the Placebo group (Table 2). Within the N-terminal domain, binding at the peptides 1–5 region suggested an interplay of IgE and IgG4, as IgE/IgG4 decreased within peptide region 1–3 while the neighboring peptide 4–5 region that includes known IgE Epitope 1 (40) had increased IgE/IgG4. Significant increases in IgE/IgG4 were observed in peptides 49 and 50, which overlap IgE Epitope 2 (GNIFSGFTPEFLEQA) (40) with sequence EFLEQA. IgE Epitope 3 (VTVRGGLRILSPDRK) (40) underwent a decrease in IgE/IgG4 at peptides 55 and 56, indicating that this IgE epitope shifted to IgG4 binding over the course of pOIT. At peptide 61, which includes the IgE Epitope 4 sequence (DEDEYEYDEEDRRRG), IgE/IgG4 increased and median SNR was >3 for both IgE and IgG4. This suggests that the flexible region of the N-terminal domain containing Epitopes 3 and 4 exhibited flanking IgG4 and IgE epitopes post-pOIT. The C-terminal domain displayed IgE/IgG4 decreases in peptides 95–97 and median SNR >3 for IgE and IgG4 in peptide 99, all of which fall within a known epitope on the flexible loop region on the periphery of the two adjacent trimers that form Ara h 3 (41).

Peanut profilin Ara h 5 is an allergen linked to pollen-associated peanut allergy (42). No peptides were significantly different in the Placebo group, and peptides 17 and 19 were significantly different in the Active group. Peptides 17 and 19 comprise a beta sheet and coil, and respectively exhibited increased IgG4 and IgE from pre to post pOIT with high effect sizes for both. This may indicate that the Ara h 5 peptide 17–19 region is an IgE epitope that experiences competition with IgG4 binding following pOIT.

Ara h 6, a 2S albumin, is a potent peanut allergen with high similarity to Ara h 2. No significant peptide shifts were identified in the Placebo group (Figure 2B). Ara h 6 peptide 5 is a linear residue on the N-terminus nearly identical to Ara h 2 (39) and was shown to have significantly lower IgE/IgG4 from pre to post-pOIT in the Active group based on paired *t*-tests (Figure 2A). Peptides 23 and 25 also had significantly higher IgG4 in the Active group from pre to post based on paired *t*-tests (Figure 2A), and these peptides constitute helix-loop-helix residue that is near-identical with Ara h 2 and forms the transition from the 4th to 5th *α*-helix (39).

Ara h 7 did not exhibit significant shifts in either the Active or Placebo group based on paired *t*-tests (Figures 2C,D), however median SNRs indicate potential epitopes. Peptides 30–32 forming the C-terminus had high effect sizes as well as IgG4 SNRs that may indicate these peptides to be IgG4 epitopes. Ara h 8, which is known as an inhaled sensitizing protein (43), is linked to oral allergy syndrome and exhibits cross-reactivity with the Bet v 1 family (44). Ara h 8 was shown to have the peptide with the highest effect size in the Active group (peptide 16), and this peptide had a significant IgE/IgG4 increase in the Active group. While median IgE and IgG4 SNRs do not suggest that peptide 16 is an epitope based on these metrics, the overlapping peptide 17 has median IgG4 SNR values indicating an epitope region (Supplementary Figure 17).

The lipid transfer protein Ara h 9 is implicated in oral allergy syndrome and is indicated as a major peanut allergen in Mediterranean populations (45, 46). Ara h 9 had 23.5% of its peptides undergo increases in IgG4 binding within the Active group (Figure 2C, Table 2), and no significant peptide shifts were identified in the Placebo group (Figure 2D). Peptides 6–7, 9, and 16 had decreased IgE/IgG4 in the Active group, while peptide 13 had increased IgE/IgG4. Peptide 16 was predicted to be a linear B-cell epitope by BepiPred 2.0 (Figure 2C). Median SNRs for Ara h 9 reveals possible IgG4 epitopes within the peptide 2–6 region, as well as peptides 9, 10, 14 and 15, all of which have been previously identified as IgE epitopes (18) and overlap with significantly-different peptides (Figure 2C, Supplementary Table 1) (18).

The oleosins Ara h 10 and 11 exhibited no significant IgE/IgG4 shifts in the Placebo group. Active group Ara h 10 peptide 12 had significantly higher IgE/IgG4 from pre to post pOIT and a large effect size (Figure 2B) decreased IgE/IgG4. Ara h 11 had significantly higher IgE/IgG4 on its N-terminus [peptide 1, (M)AEALYY], and peptides 16–20, 22, and 23 suggest an IgE binding region that is flanked by an IgG4 binding region (Figure 2B). Two regions in Ara h 11 comprised of peptides 5–6 and 16–18 exhibited significant IgE/IgG4 shifts. Peptides 5–6 respectively showed increased and decreased IgE/IgG4 on either side of this overlapping peptide, while IgE/IgG4 increased in the peptide 16–18 region.

### 3.3. Peptide pre-vs.-post pOIT correlations and supervised binary classification

**Figure 3:**
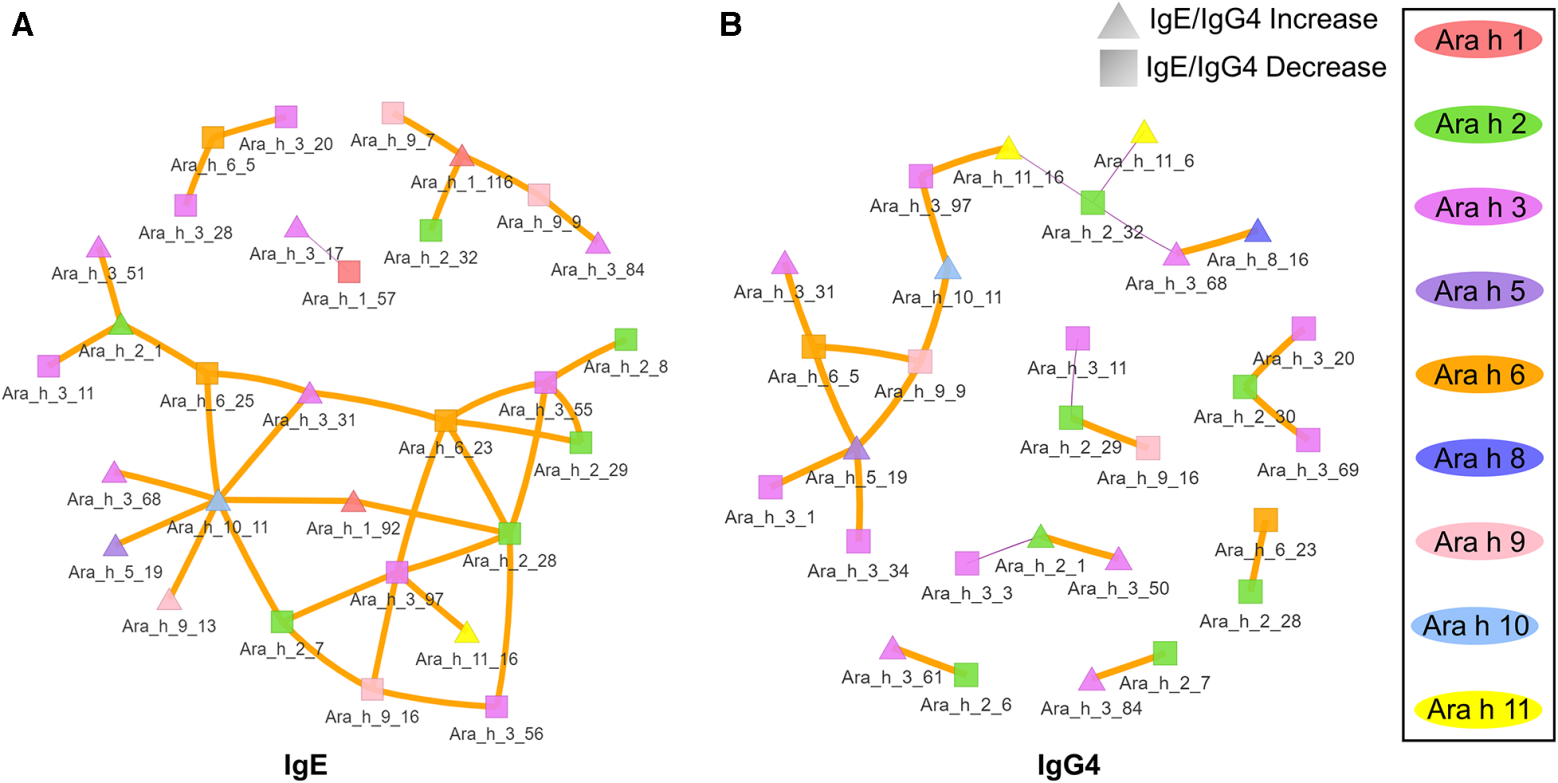
Correlation network of Ara h peptides for active group participants. (**A**) IgE and (**B**) IgG4 Pearson correlations were performed for peptides found to have significantly different within-treatment IgE/IgG4 from pre-to-post pOIT based on paired *t*-tests. Orange edges indicate Pearson correlation coefficients >0.7, and purple edges indicate coefficients <-0.7. The width of the edges indicates a greater absolute value of the correlation coefficient. Square nodes indicate a peptide with significantly decreasing IgE/IgG4 ratio (increasing IgG4), and triangular nodes indicate increasing IgE/IgG4 ratio (increasing IgE) based on paired *t*-test results.

Given the assumption that pre- and post-pOIT sera epitope binding patterns represent a snapshot of a dynamic system, we wished to identify relationships of immunoglobulin binding intensity among individual allergen epitopes that occurred over the course of the study. Pearson correlation networks were constructed to highlight IgE and IgG4 binding relationships of Ara h peptides exhibiting significant IgE/IgG4 shifts over the course of pOIT. The IgE binding intensity for major Ara h 2 epitopes were shown to be positively correlated with known and newly identified epitopes of other peanut proteins (Figure 3A). IgE binding for Ara h 2 peptide region 28–29 was shown to be positively correlated with Ara h 6 peptide 23 (Figure 3A), all of which shifted towards decreased IgE/IgG4 post pOIT and exhibited high Hedges' *g* effect sizes (>0.8) (Figure 2A). The Ara h 2 peptide 28–29 region exhibited weaker positive IgE correlations with Ara h 3 peptides 55, 56 (Ara h 3 Epitope 3) and 97 [C-terminal region flexible loop epitope (41)]. The known Ara h 2 epitope peptide 7 had a positive IgE correlation with Ara h 3 peptide 97. Ara h 2 peptides 8 and 9 have positive IgE correlation coefficients with Ara h 9 peptide 2 (Figure 3A). Ara h 2 peptide 9 is a known epitope, and Ara h 9 peptide 2 is a candidate major IgE and IgG4 epitope based on median SNR (Supplementary Figure 18). Ara h 3 had strong positive IgE and IgG4 correlations with multiple Ara h 2 IgE epitopes and significantly different Ara h 6 peptides (Figures 3A,B). Ara h 5 peptide 19 was shown to have positive IgG4 correlation coefficients with Ara h 3, Ara h 6, and Ara h 9 peptides that exhibited decreases in IgE/IgG4 (Figure 3B). This may indicate that this Ara h 5 IgE epitope undergoes antibody binding shifts in concert with these other peptides, and that it may be an important peptide for monitoring tolerance due to its high effect size (Figure 2A). Ara h 10 peptide 11 was found to have positive IgE correlations with Ara h 3, Ara h 5, and Ara h 9 peptides that displayed increased IgE/IgG4 at the population level, along with Ara h 6 and Ara h 2 peptides whose IgE/IgG4 decreased (Figure 3A).

**Figure 4:**
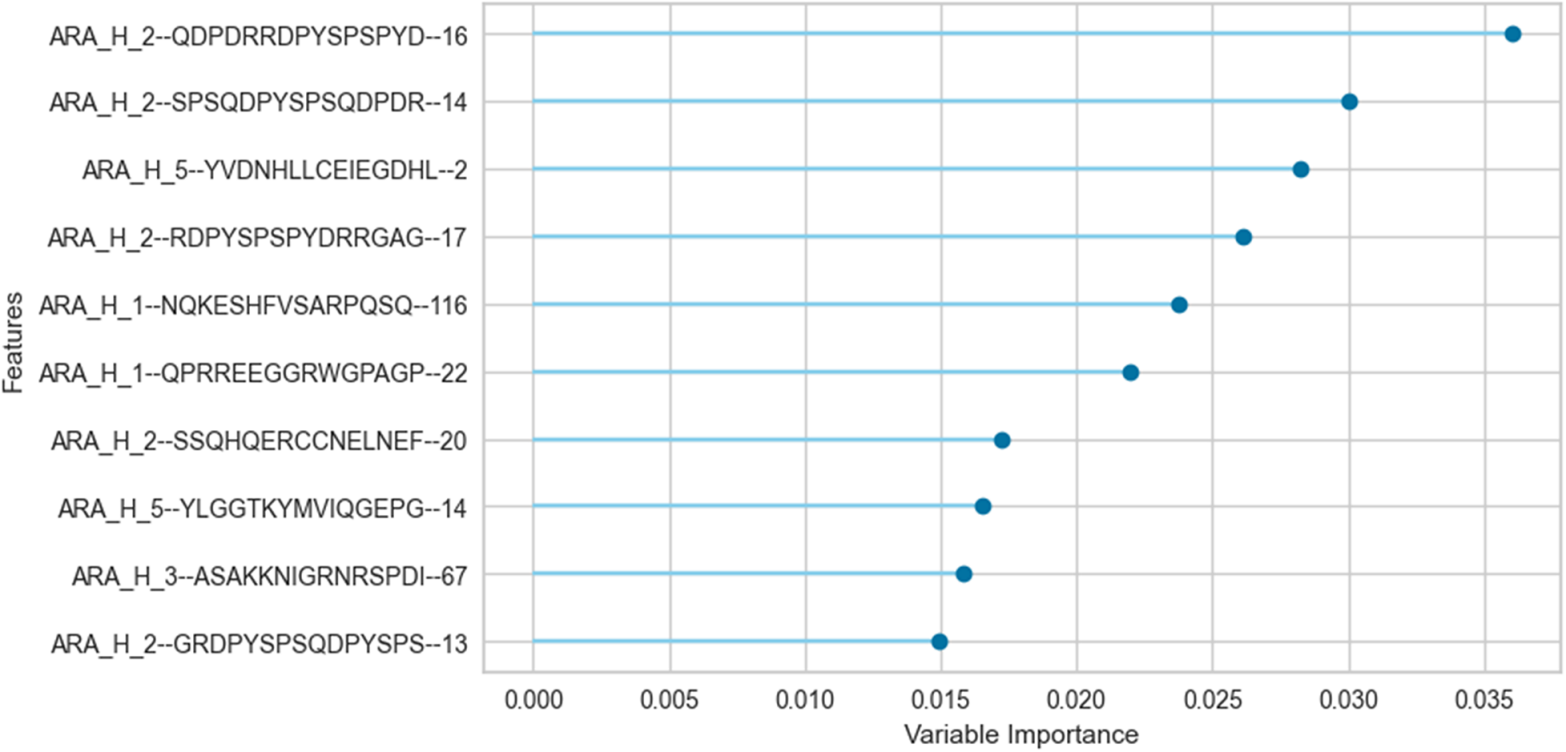
Important peptide features for the extremely randomized trees binary classifier in predicting desensitization. The peptide features that contribute most to the scoring of this particular pSLIT-trained ERT classifier to predict desensitization were identified with Gini Impurity feature importance and are shown from top to bottom on the y-axis in the order of decreasing importance.

We trained an ERT classifier to predict participant desensitization to peanut (Table 3) and used Gini Impurity feature importance to identify peptides that were most important to this particular model (Figure 4). Peptides from Ara h 1 (peptides 116, 22), Ara h 2 (peptides 16, 14, 17, 20, 13), Ara h 3 (peptides 67) and Ara h 5 (peptides 2, 14)), comprised the top 10 most important features for the ERT classifier (Figure 4). Ara h 2 peptides 16 and 14 were the two most important features for the finalized model, followed by predicted IgE epitope Ara h peptide 2 and known IgE epitopes Ara h 2 peptide 17, Ara h 1 peptide 116 and Ara h 1 peptide 22 (Figure 4). Ara h 2 peptide 20, Ara h 5 peptide 14, Ara h 3 peptide 67, and known IgE epitope Ara h 2 peptide 13 comprised the next tier of important features (Figure 4).

**Table 3:** Performance metrics for top 10 finalized binary classifier models trained on pSLIT and predicting desensitization on unseen PALISADE dataset.

Model	Session	Acc	AUC	Recall	Prec	F1	Kappa	MCC
Voting classifier	classify-2_seed-93	0.8235	0.8235	0.7059	0.9231	0.8	0.6471	0.6658
**Extra trees classifier**	**classify-0_seed-388**	**0.8333**	**0.8756**	**0.6667**	1	**0.8000**	**0.6667**	**0.7071**
Extra trees classifier	classify-1_seed-215	0.7941	0.8339	0.7647	0.8125	0.7879	0.5882	0.5893
Extra trees classifier	classify-1_seed-692	0.8235	0.8581	0.6471	1	0.7857	0.6471	0.6916
Random forest classifier	classify-2_seed-866	0.8235	0.8304	0.6471	1	0.7857	0.6471	0.6916
SVM - linear kernel	classify-1_seed-677	0.7941	0.7941	0.7059	0.8571	0.7742	0.5882	0.5976
Voting classifier	classify-1_seed-743	0.7941	0.7578	0.7059	0.8571	0.7742	0.5882	0.5976
Decision tree classifier	classify-0_seed-730	0.7353	0.7924	0.8824	0.6818	0.7692	0.4706	0.4924
SVM - linear kernel	classify-1_seed-518	0.7647	0.8131	0.7647	0.7647	0.7647	0.5294	0.5294
SVM - linear kernel	classify-2_seed-77	0.7647	0.7855	0.7647	0.7647	0.7647	0.5294	0.5294
Voting classifier	classify-0_seed-539	0.7941	0.8478	0.6471	0.9167	0.7586	0.5882	0.6155
Voting classifier	classify-1_seed-293	0.7941	0.7941	0.6471	0.9167	0.7586	0.5882	0.6155

Sessions labeled as classify 0, 1, and 2 indicate experiments using FST of 0.6, 0.7, and 0.8, respectively. Bold text indicates the selected model used in this study (Extra Trees Classifier, classify-0_seed-388). Acc, accuracy; AUC, area under the receiver operating characteristic curve; Prec, precision; MCC, Matthews correlation coefficient.

## 4. Discussion

This study utilized sera from peanut-allergic individuals undergoing pOIT with PTAH to identify linear peptide-based IgE and IgG4 epitope shifts of Ara h 1, 2, 3, 5, 6, 7, 8, 9, 10 and 11, as well as shifts in IgE binding to intact Ara h 1, 2, 3, 6, 8, and 9 allergens. A larger set of peanut proteins and peptides were assessed in this study compared to most previous pOIT studies, and specific epitope shifts in participants undergoing pOIT using a standardized, FDA-approved treatment were highlighted. In comparison to the Placebo treated, the Active group population exhibited a greater number of significant shifts in IgE epitopes binding to IgG4 after 12 months of pOIT, as seen in previous pOIT studies (6, 14, 47). In 9 of the 15 (60%) Active group participants, the peanut-specific (ps) IgE and total IgE was increased or relatively unchanged after 12 months of pOIT, while the MWD following SPT decreased in 14 of 15 (93.3%-100%) participants (exit MWD was not measured for participant 004-007) (Table 1). However, 11 (73.3%) of the Active group participants exhibited relative increases in psIgG4 ranging from 33.3% to 9,244% following 12 months of pOIT. This indicates that at the 12-month point, the total and ps-IgE for a majority of the subjects has not started to decrease in response to treatment, and that the shifts in IgE/IgG4 of the peptides are more likely due to the increase in IgG4 than decrease in IgE. Therefore, it appears that the rise in IgG4 levels during pOIT may be more important than the decrease in psIgE to modulating desensitization, as evident by maximum tolerated dose (MTD, Table 1) at 12 months. The post-pOIT decrease in participant IgE binding to intact proteins in ISAC immunoassays may be due in part to inhibition of IgE binding caused by the increased presence of competing IgG4 after immunotherapy (47–49), which may better reflect the *in vivo* state of a participant's allergen sensitivity (48, 50).

In addition to overall IgE and IgG4 levels in patients, this study offers insight into antibody binding shifts for individual proteins and peptides that occur over the course of pOIT. At a population level, the IgE and IgG4 epitopes overlapped and the IgE epitopes showed limited changes with 12 months of pOIT. The stability of IgE epitopes through allergen-specific immunotherapy or vaccination has previously been shown at an individual level in the case of inhaled allergens (51). Another study using the same grass pollen allergen microarrays found that allergen immunotherapy induced expanded IgG-type and IgE antibodies against novel epitopes after one year of treatment compared to untreated controls (52). Ara h 1, 2, 3, and 6 are currently accepted as the four major peanut allergens (53), however it was surprising that the PALISADE participant group did not exhibit significant population-level IgE binding changes to intact Ara h 1. It appears that ISAC Ara h 1 IgE intensity increases in some participants (e.g., participants 062-014 and 035-013) or does not shift at all (participant 062-017) (Figure 1B). Interestingly, participants 062-014 and 035-013 both had a high psIgE/total IgE ratio and were able to reach a MTD of 600 mgs at the 12-month exit timepoint (Table 1). Ara h 2 IgE binding intensity increased after pOIT in these two participants, and their sera IgE did not bind to Ara h 3 (Figure 1B). Meanwhile, 25 of 124 Ara h 1 peptides showed a decrease in IgE/IgG4 with only 3 peptides having increased IgE/IgG4 with high effect size (Figure 2A). It is possible that 2 of these Ara h 1 peptides (107 and 116) are important in conformational epitopes that drive the increased IgE binding to intact Ara h 1 on the ISAC array for certain participants. Ara h 6 shares 60% sequence identity and multiple IgE epitopes with Ara h 2, resulting in a degree of cross-reactivity and the common occurrence of peanut-allergic individuals producing IgE against Ara h 2 and 6 (54). This likely explains why the Active group had significant reductions and comparable intensity values for ISAC IgE binding to Ara h 2 and Ara h 6 from pre- to post-pOIT. In addition, the Ara h 2 IgE epitope spanning peptides 28 and 29 was shown to be an IgE and IgG4 epitope based on median SNR (Supplementary Figure 12) and has positive IgE correlations with Ara h 6 peptide 23 (Figure 3A), which was identified as an IgE and IgG4 epitope (Supplementary Figure 15) and contains a portion of a highly homologous epitope to Ara h 2 IgE epitope 7 (KRELRMLP) (19).

Aside from shifts in antibody binding to major peanut allergens, this study also highlights shifts in binding to minor allergens Ara h 5, 7, 8, 9, 10, and 11 over the course of pOIT. Ara h 5 peptide 19 was shown to have positive IgG4 correlation coefficients >0.7 with Ara h 3, Ara h 6, and Ara h 9 peptides (Figure 3B). Interestingly, the sensitizing protein Ara h 5 was found to contribute important features (peptides 2 and 14) for the ERT classifier predicting peanut desensitization (Figure 4). These Ara h 5 peptides were not found to be significantly different based on paired *t*-tests, however they were indicated to be IgE epitopes based on median SNR (Supplementary Figure 14). Given the potential role of Ara h 5 as a sensitizing protein for peanut allergy due to its similarity to cross-reactive proteins such as Bet v 2 (birch pollen) and Hev b 8 (latex profilin) (55), gaining a better understanding of Ara h 5's relationship to other peanut allergens with regards to pOIT may indicate its use as a marker for tolerance. Ara h 7 did not have significantly different IgE or IgG4 binding in Active participant sera following pOIT, as opposed to a previous pOIT study (PRROTECT) where participants received omalizumab (56). It is possible that the concurrent 19-week omalizumab treatment with pOIT may have resulted in increased IgG binding to Ara h 7 in PRROTECT sera (56), vs. non-significant changes in binding in the PALISADE study. However, median SNRs for Ara h 7 peptides 4, 15, 21, and 26 identify them as IgE and IgG4 epitopes in this study. This study also explores IgE and IgG4 binding shifts in the oleosins Ara h 10 and 11, which have not been studied with regards to pOIT. This study reveals the presence of IgE and IgG4 binding regions with high effect sizes, indicating that they are not only statistically significant but the magnitude of this binding difference over the course of pOIT lends to the potential importance of these regions in applied peanut allergy diagnostics. Further studies are needed to determine the roles of these oleosin binding regions in peanut allergy and tolerance.

The notion of major allergen epitope regions shifting antibody binding in concert with sensitizing proteins is supported by Ara h 9 peptide links to major allergens. Positive correlations >0.7 were seen for of Ara h 1 peptide 116 with Ara h 9 peptides 7 and 9 (IgE, Figure 3A), Ara h 2 peptide 29 with Ara h 9 peptide 16, and Ara h 6 peptide 5 with Ara h 9 peptide 9 (IgG4, Figure 3B). The positive IgE correlations of Ara h 9 peptides with known major allergen IgE epitopes and epitopes important in predicting desensitization (Ara h 1 peptide 116, Figure 4), along with these peptides experiencing a shift towards increased IgG4 binding, highlights that Ara h 9 may have greater roles in the development and treatment of allergy given that it is a major allergen in Mediterranean populations (18, 45, 46). Ara h 9 peptide 2 was a major epitope for the PALISADE participant pool based on median SNRs (Supplementary Figure 18), and previous studies have shown US participant sera to be IgE reactive to this peptide as well as other previously described binding regions (18). The US participants described in this study did not exhibit IgE binding to the intact Ara h 9 proteins, in line with ISAC results of US patients described in Kronfel et al. (18). Further research is required to determine whether binding to linear Ara h 9 epitopes influences sensitivity to major allergens in US patients.

The oleosins Ara h 10 and Ara h 11 are understudied with regards to peanut allergy, and this study provides early insight into potential roles that these proteins may play in allergy and development of tolerance to peanuts. Besides identifying epitopes and potential biomarkers for pOIT, correlation networks show a first glance into the links these proteins may have with better-studied allergens. For instance, Ara h 10 and Ara h 11 have positive IgE and IgG4 correlation coefficients with peptides in Ara h 1, 2, 3, 5, 6, 8, and 9 (Figure 3). The presence of peptides in Ara h 10 and Ara h 11 with significantly different IgE/IgG4 shifts and high effect sizes (Figure 2C) suggest that these could be important regions in understudied proteins that have applications towards allergy diagnosis and determination of tolerance after pOIT.

This study utilized an ERT binary classifier to predict participant desensitization to peanut and we used Gini Impurity feature importance to identify the most important peptide features for this particular model's classification task (Figure 4). This method identified peptide features that were significantly different based on between-group (LM) and within-group (paired *t*-test) IgE/IgG4 comparisons (Figure 2, Figure 4). Ara h 2 peptides 13, 14, 16, and 17 were identified as top 10 important features for the ERT model while being significantly different in the Active vs. the Placebo group after the course of pOIT based on an LM. While Ara h 2 peptides 13, 14 and peptide 16 should be interpreted with some caution given their higher adjusted p values (*p* = 0.1, 0.1, and 0.059, respectively) versus Ara h 2 peptide 17 (*p* = 0.046), this does support the finding that these peptides were important for predicting desensitization since they showed significantly lower IgE/IgG4 (increased IgG4 and/or decreased IgE) in the Active treatment group, which underwent pOIT and became desensitized, vs. the Placebo group, which did not. While machine learning algorithms can be very powerful tools, the limited number of participant sera obtained for the PALISADE study and necessity of training the model on a larger pSLIT dataset may be responsible for the identification and some inconsistencies in important peptides compared to paired *t*-tests. Future studies will require larger amounts of training data to increase model performance.

Increasing our understanding of which epitopes shift from IgE to IgG4 binding over the course of oral immunotherapy is vital towards developing a framework for identifying successful pOIT treatments, as well as improving potential peptide-based diagnostic methods for food allergy. It is also important to note that the binding of IgE and IgG4 to extracts, components, or allergen peptides can diverge and that each level of binding may contribute important information regarding the state of immune modulation and subject response to types and duration of treatments. Future studies with more timepoints and clinical data regarding other allergies will aid in determining long-term shifts in IgE and IgG4 epitope binding once participants reach a maintenance phase. This study contributes to our growing knowledge of peanut allergy dynamics over the course of oral immunotherapy, demonstrates which epitopes shift for participants taking PTAH, and provides an initial framework for identifying critical epitopes that determine if peanut-allergic participants have reached desensitization after treatment.

## Data Availability

The original contributions presented in the study are included in the article/**Supplementary Material**, and further inquiries can be directed to the corresponding author.
