# Supplementary material for: IgE and IgG4 epitopes of the peanut allergens shift following oral immunotherapy
Source: Front Allergy. 2023 Nov 29;4:1279290. doi: 10.3389/falgy.2023.1279290 (PMC10717846; doi:10.3389/falgy.2023.1279290)
Supplement: Supplementary file 1 [file Datasheet1.pdf]

# *Supplementary Material*

**Supplementary Table 1. Synthetic linear peptides for Ara h proteins used in this study.** These 15-mer peptides are offset by 5 amino acids and represent the entire peptide sequence of each Ara h protein.  (Attached Excel file)

**Supplementary Table 2. Linear regression model fit metrics for Ara h peptides used in this study.** Metrics include mean squared error, multiple $R^2$, and adjusted $R^2$. (Attached Excel file)

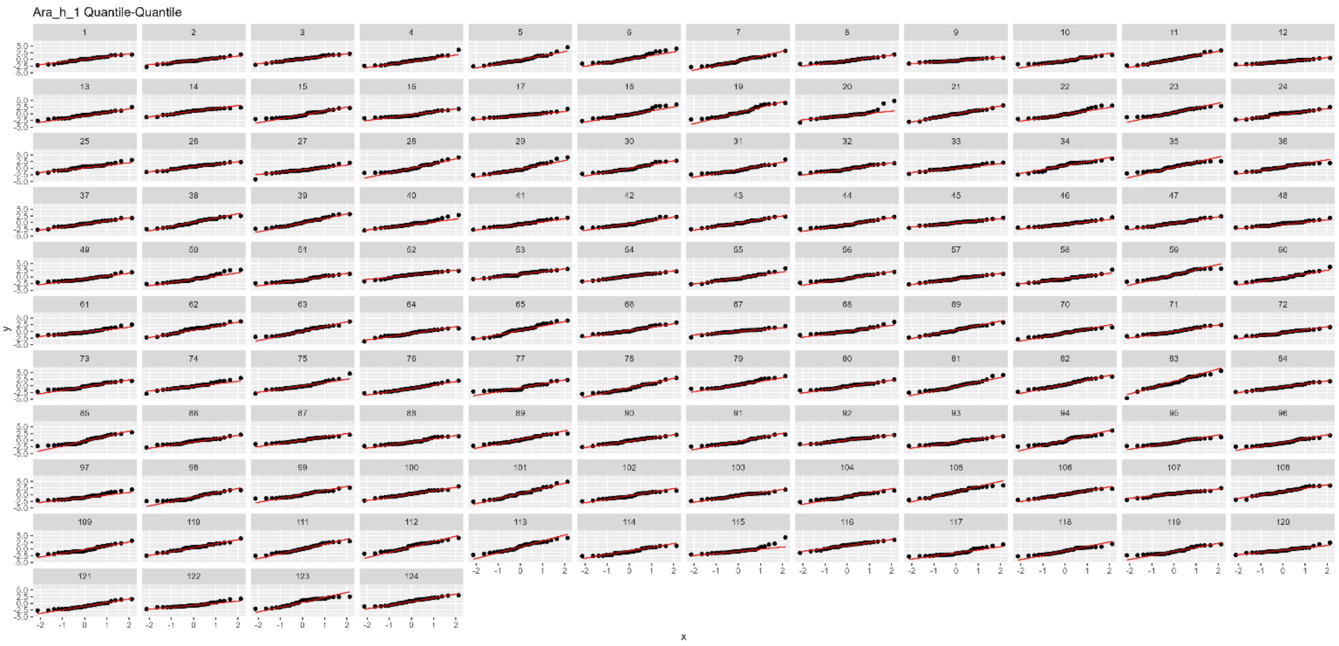


**Supplementary Figure 1. Quantile-quantile plots for Ara h 1 peptide log2 transformed IgE/IgG4 ratios.**

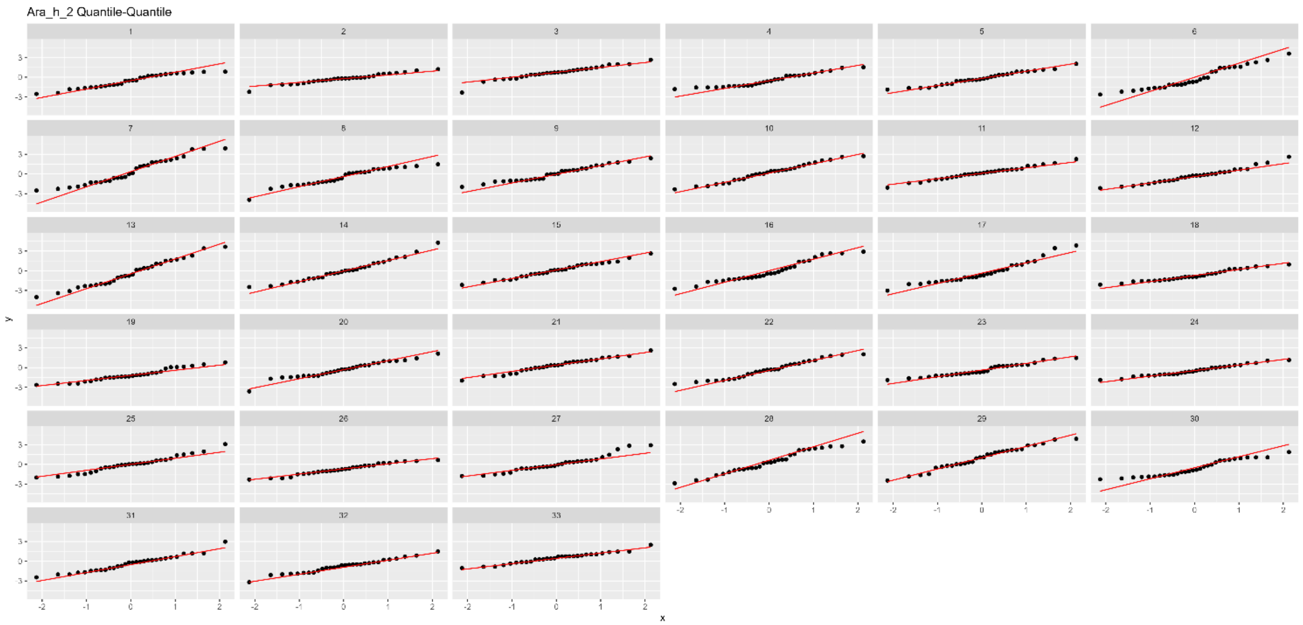


**Supplementary Figure 2. Quantile-quantile plots for Ara h 2 peptide log2 transformed IgE/IgG4 ratios.**

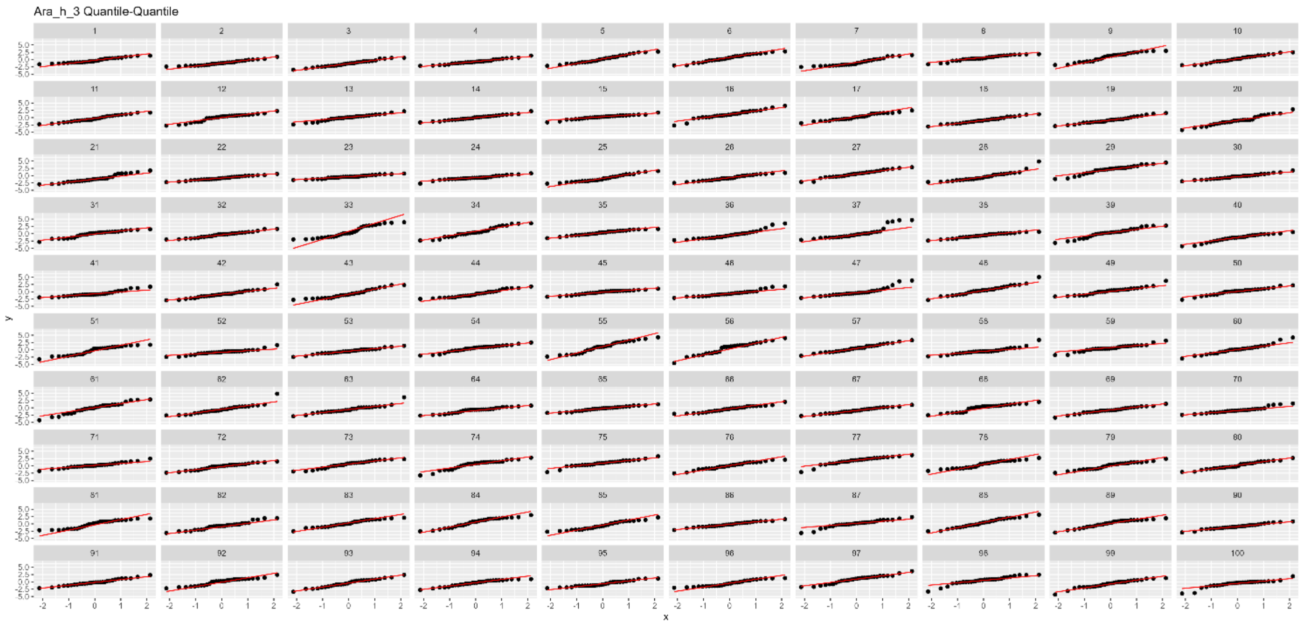


**Supplementary Figure 3. Quantile-quantile plots for Ara h 3 peptide log2 transformed IgE/IgG4 ratios.**

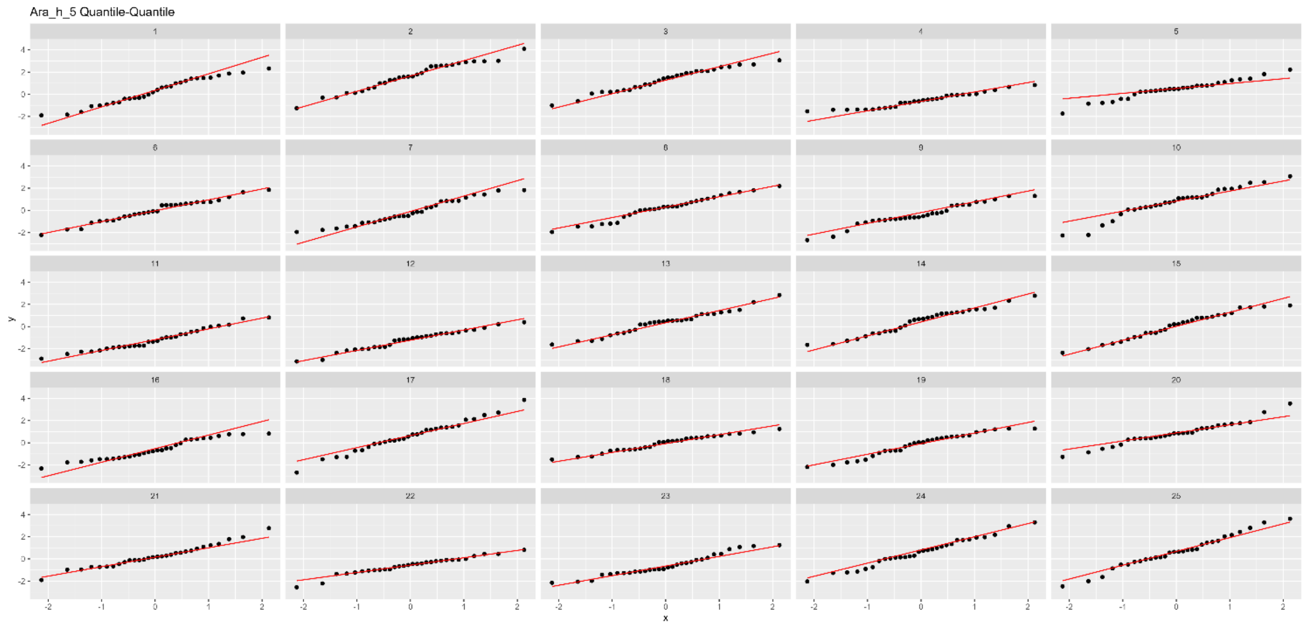


**Supplementary Figure 4. Quantile-quantile plots for Ara h 5 peptide log2 transformed IgE/IgG4 ratios.**

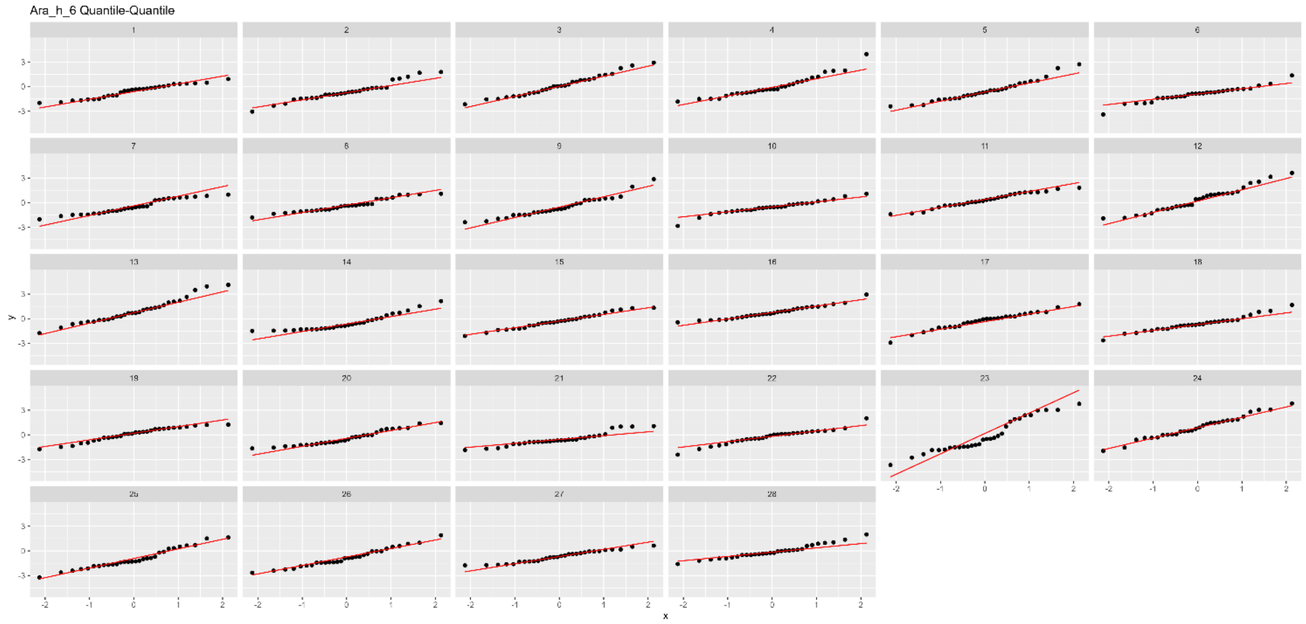


**Supplementary Figure 5. Quantile-quantile plots for Ara h 6 peptide log2 transformed IgE/IgG4 ratios.**

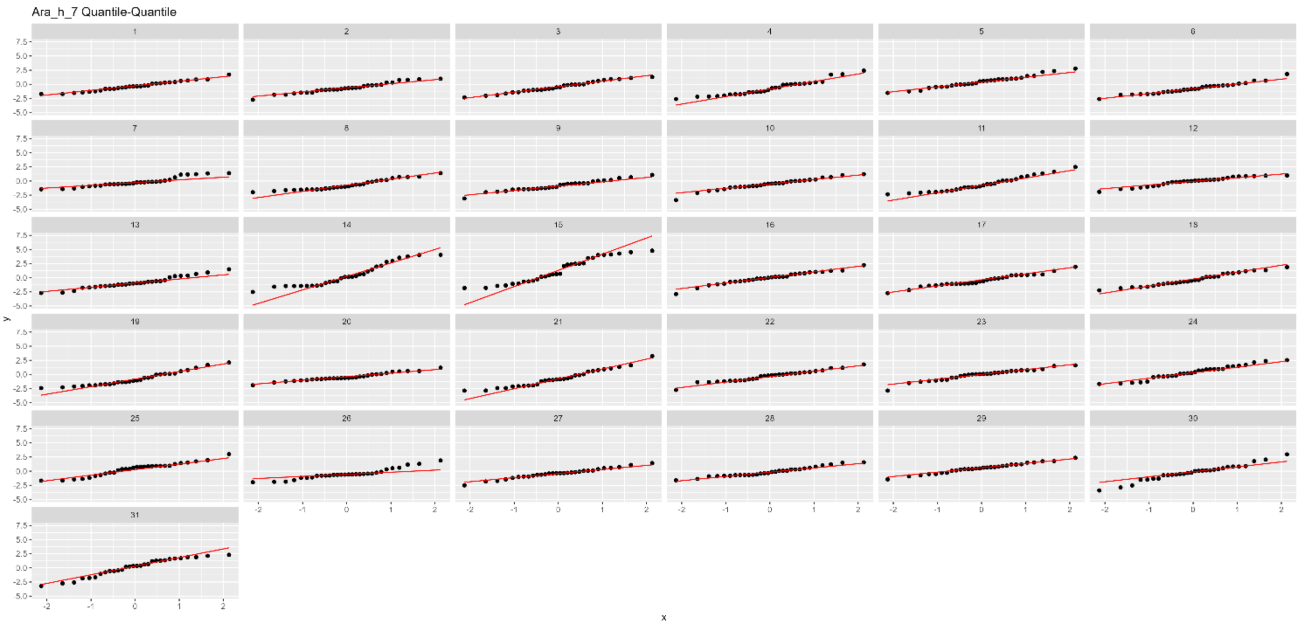


**Supplementary Figure 6. Quantile-quantile plots for Ara h 7 peptide log2 transformed IgE/IgG4 ratios.**

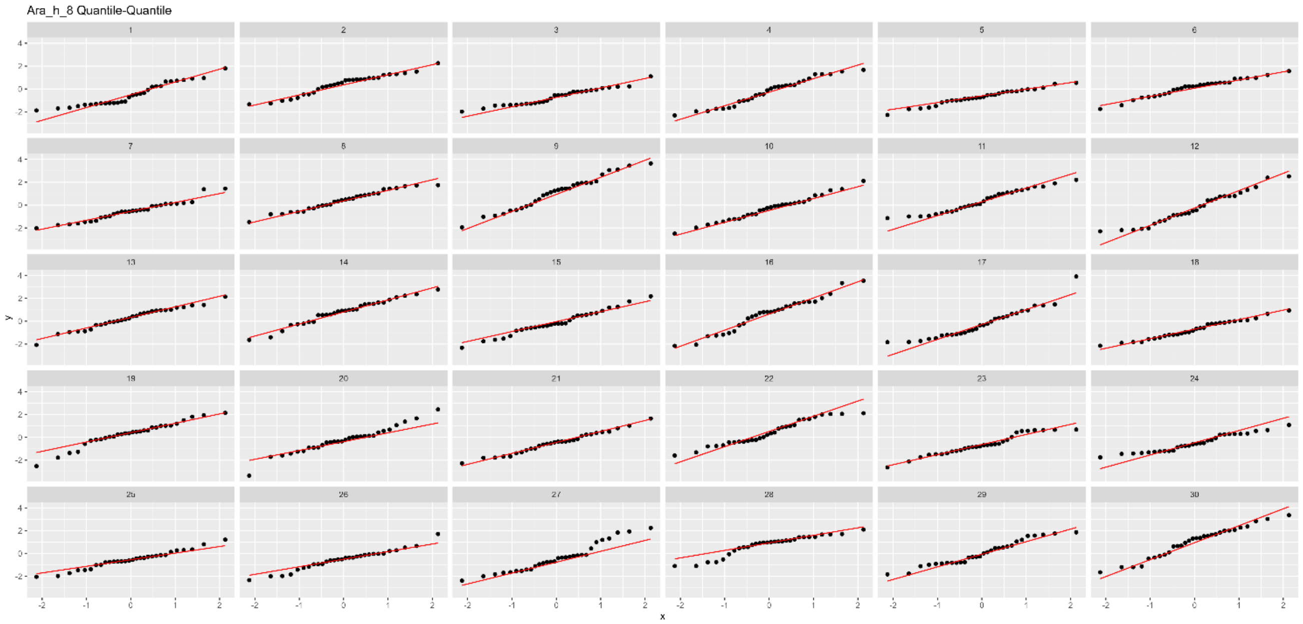


**Supplementary Figure 7. Quantile-quantile plots for Ara h 8 peptide log2 transformed IgE/IgG4 ratios.**

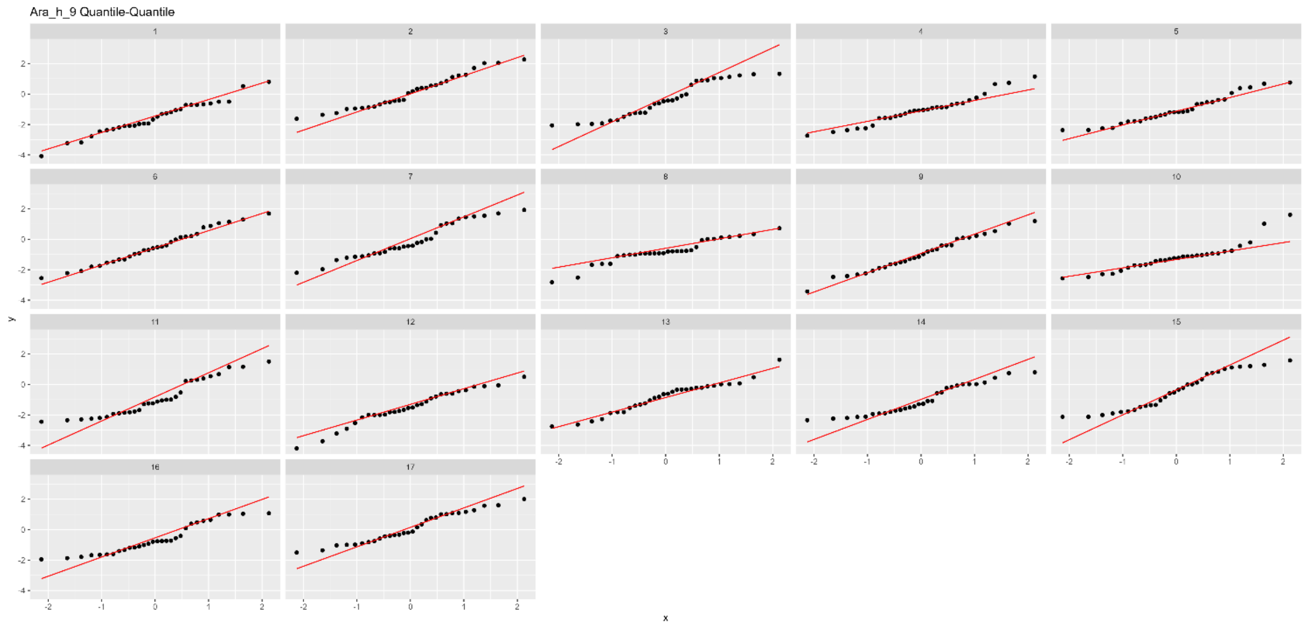


**Supplementary Figure 8. Quantile-quantile plots for Ara h 9 peptide log2 transformed IgE/IgG4 ratios.**

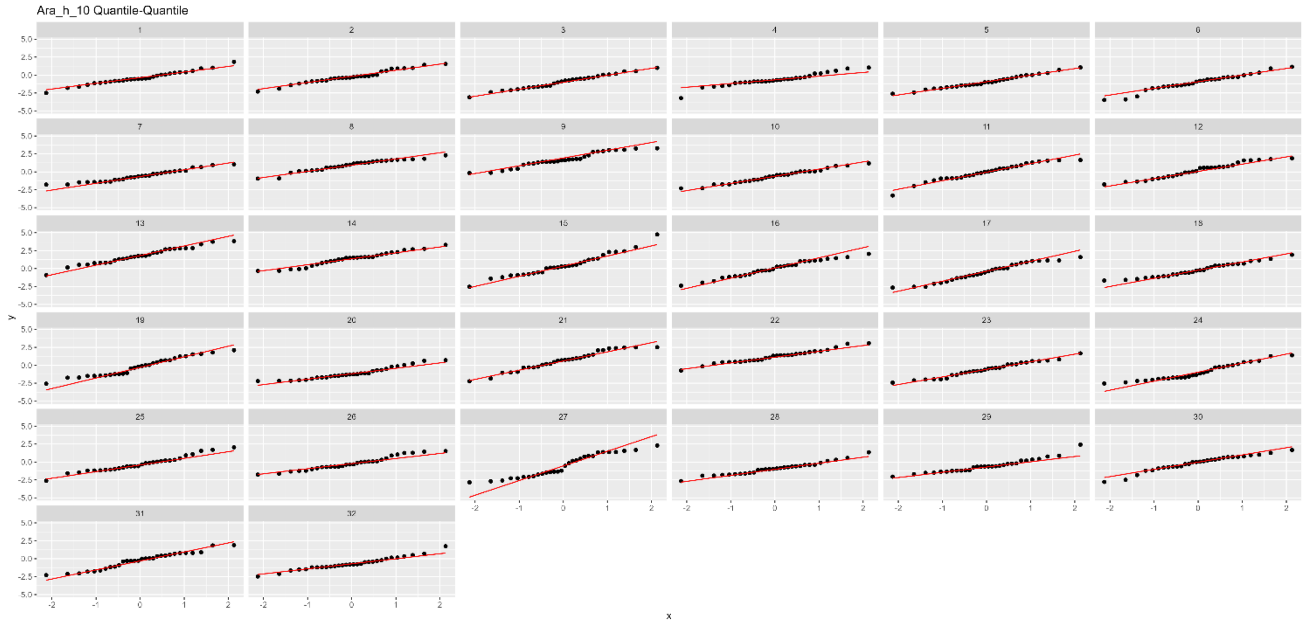


**Supplementary Figure 9. Quantile-quantile plots for Ara h 10 peptide log2 transformed IgE/IgG4 ratios.**

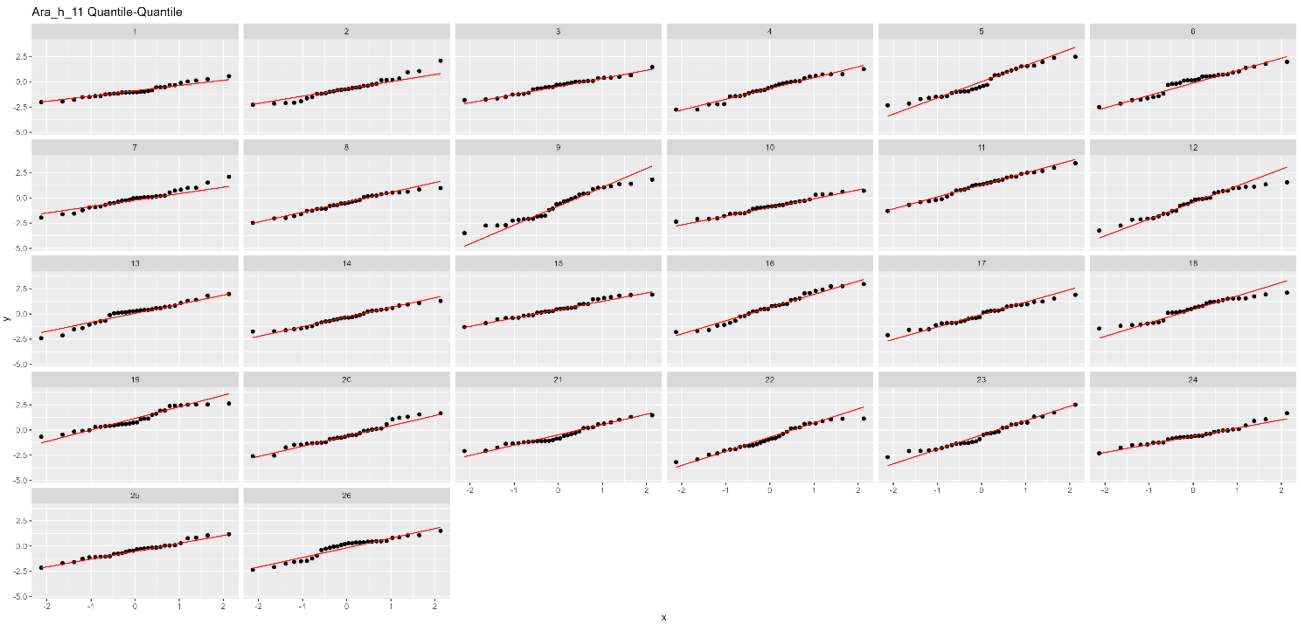


**Supplementary Figure 10. Quantile-quantile plots for Ara h 11 peptide log2 transformed IgE/IgG4 ratios.**

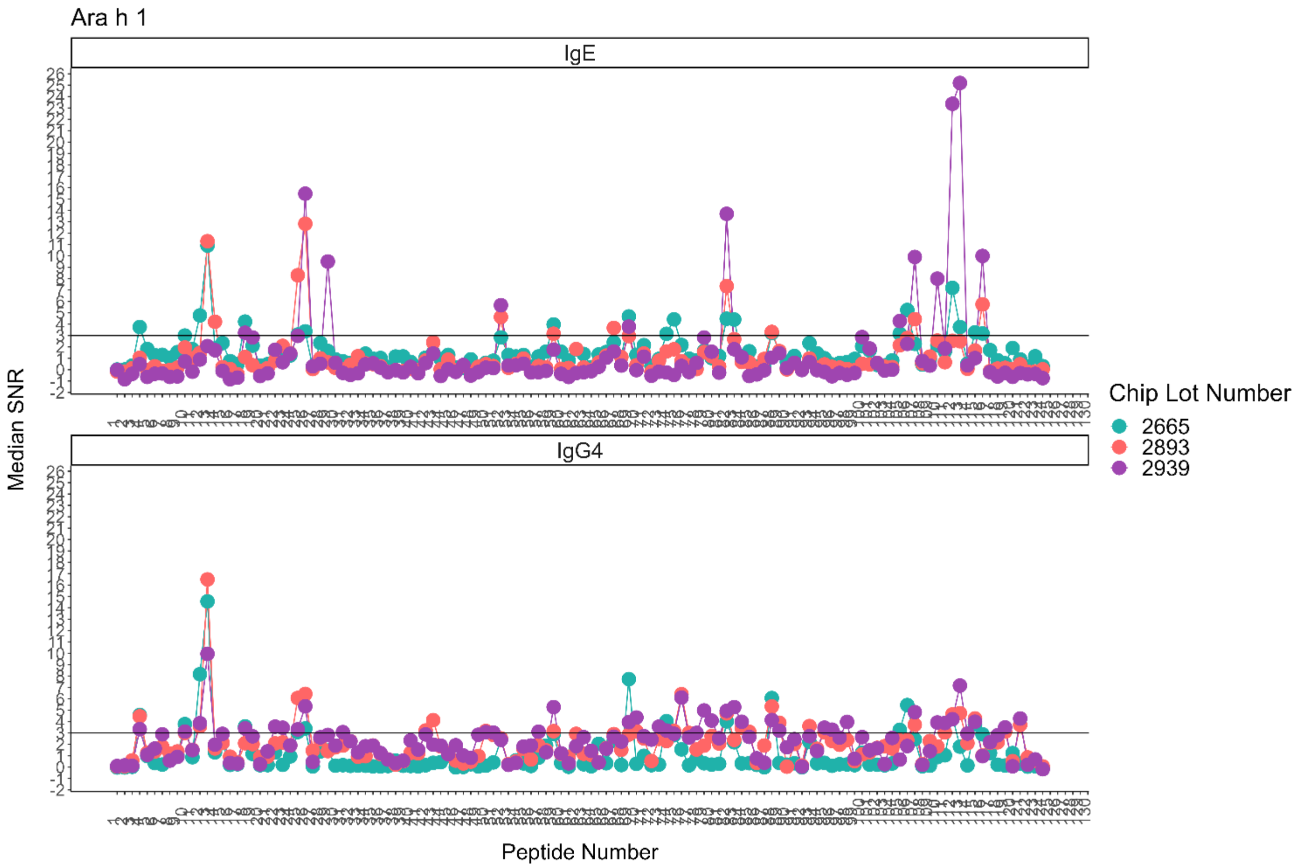


**Supplementary Figure 11. IgE and IgG4 signal to noise (SNR) ratios for Ara h 1, phase 3 pOIT 3 pOIT participants.** Major epitopes are indicated by peptides with median SNR > 3 for >50% of participants.

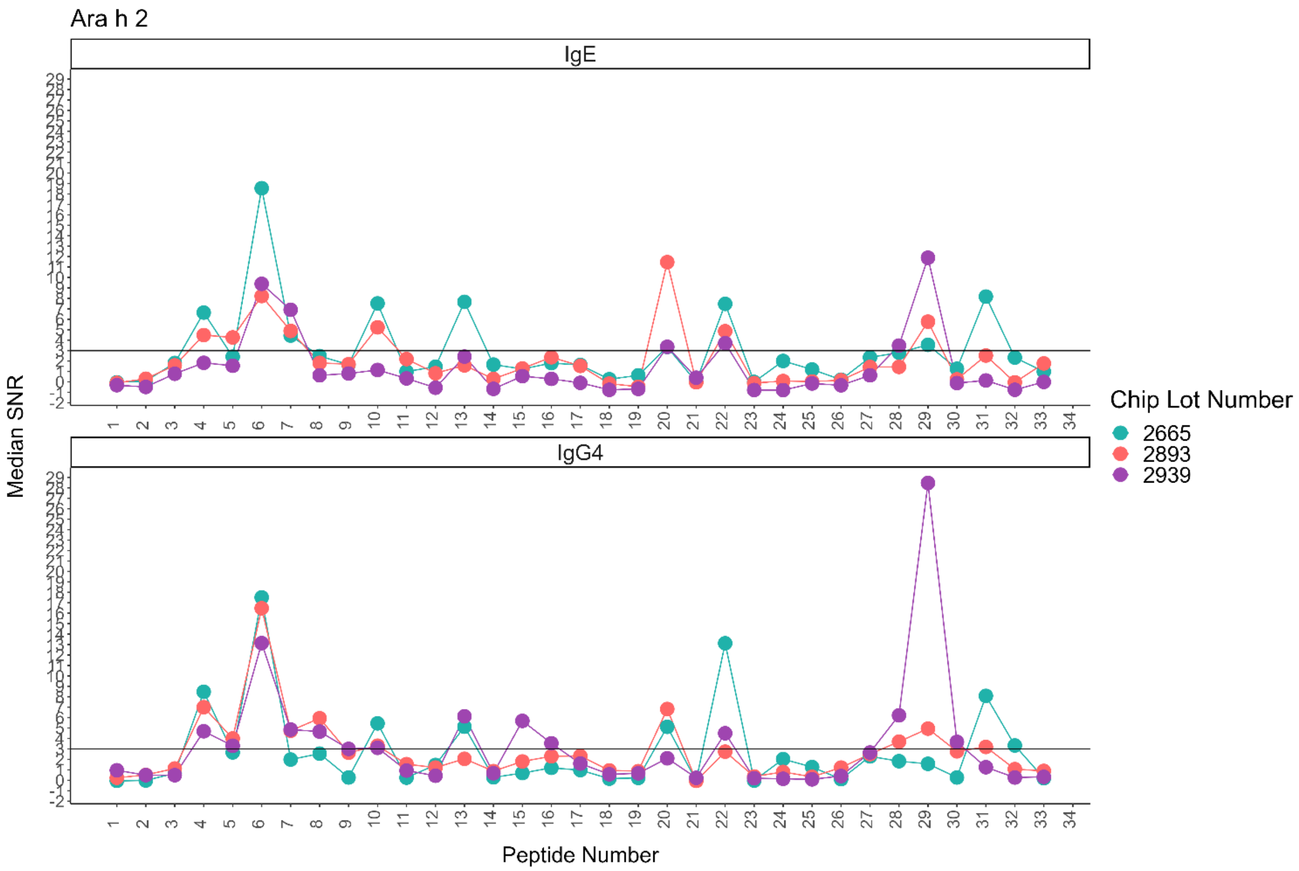


**Supplementary Figure 12. IgE and IgG4 signal to noise (SNR) ratios for Ara h 2, phase 3 pOIT participants.** Major epitopes are indicated by peptides with median SNR > 3 for >50% of participants.

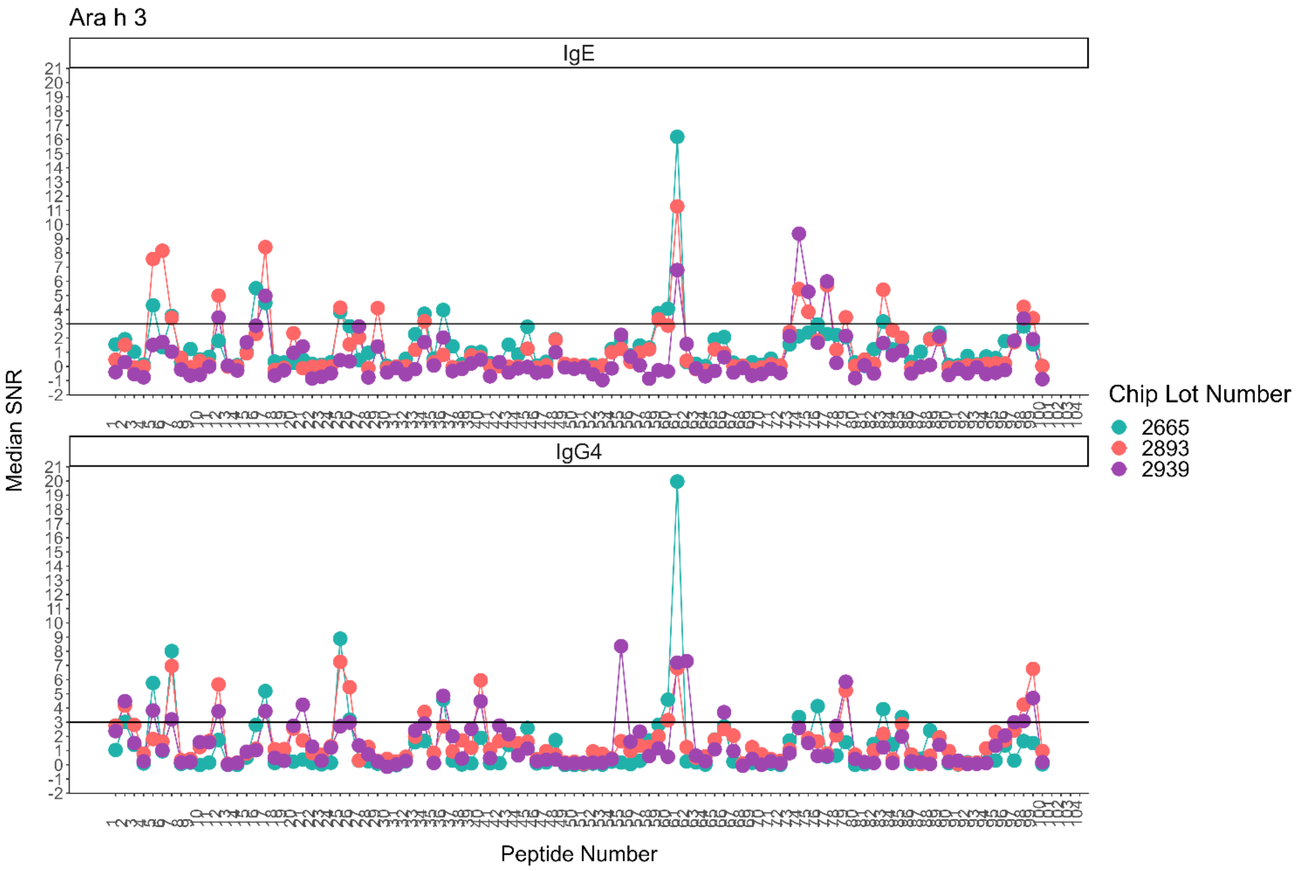


**Supplementary Figure 13. IgE and IgG4 signal to noise (SNR) ratios for Ara h 3, phase 3 pOIT participants.** Major epitopes are indicated by peptides with median SNR > 3 for >50% of participants.

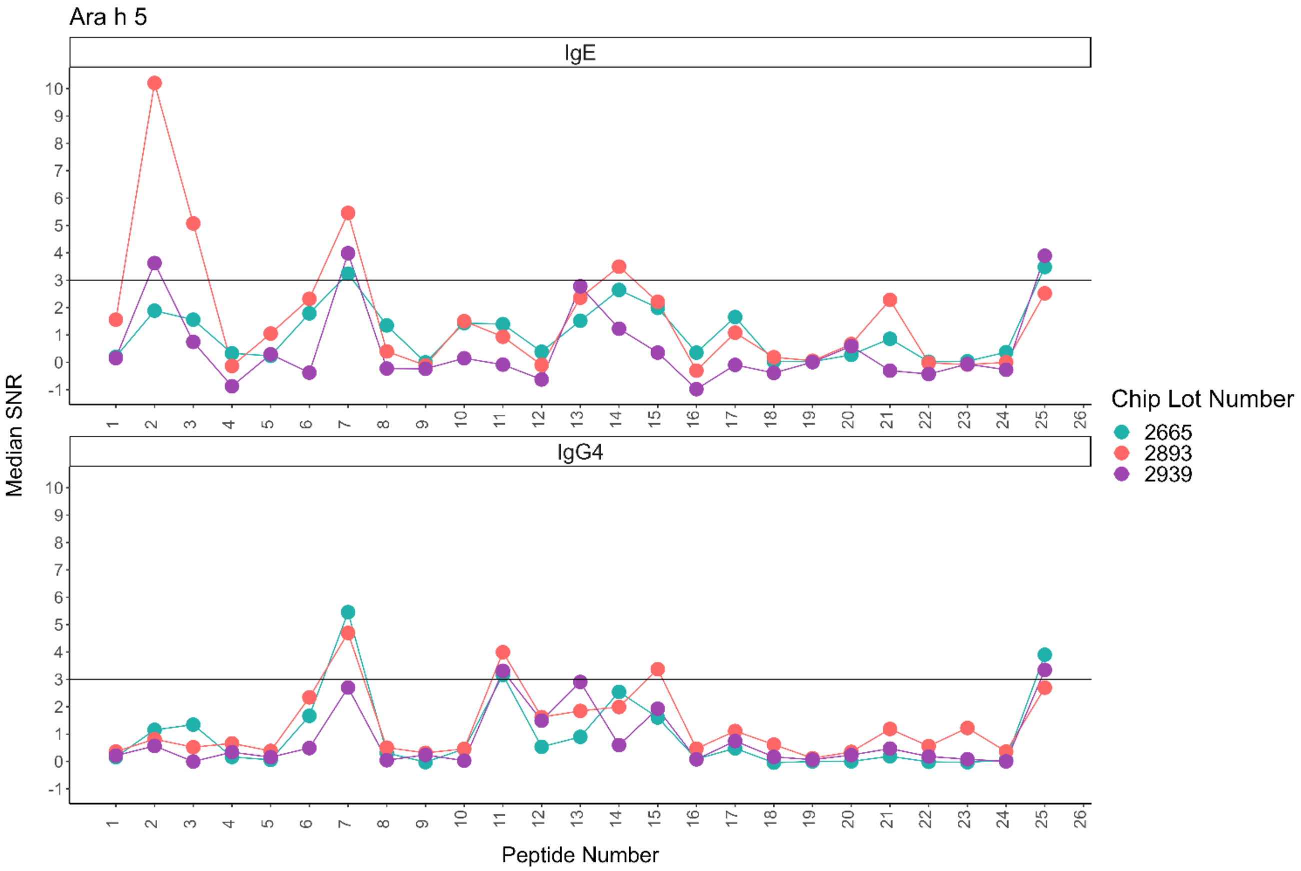


**Supplementary Figure 14. IgE and IgG4 signal to noise (SNR) ratios for Ara h 5, phase 3 pOIT participants.** Major epitopes are indicated by peptides with median SNR > 3 for >50% of participants.

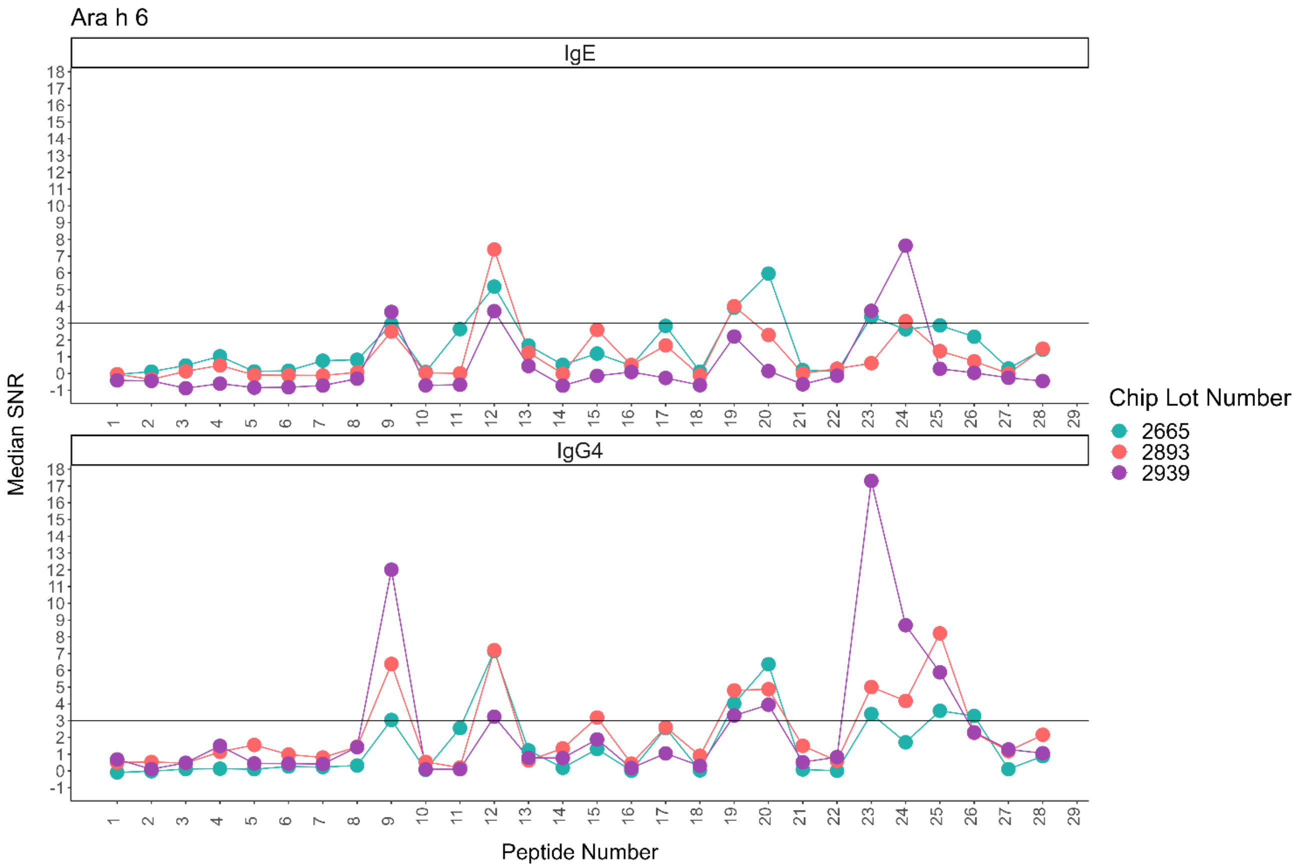


**Supplementary Figure 15. IgE and IgG4 signal to noise (SNR) ratios for Ara h 6, phase 3 pOIT participants.** Major epitopes are indicated by peptides with median SNR > 3 for >50% of participants.

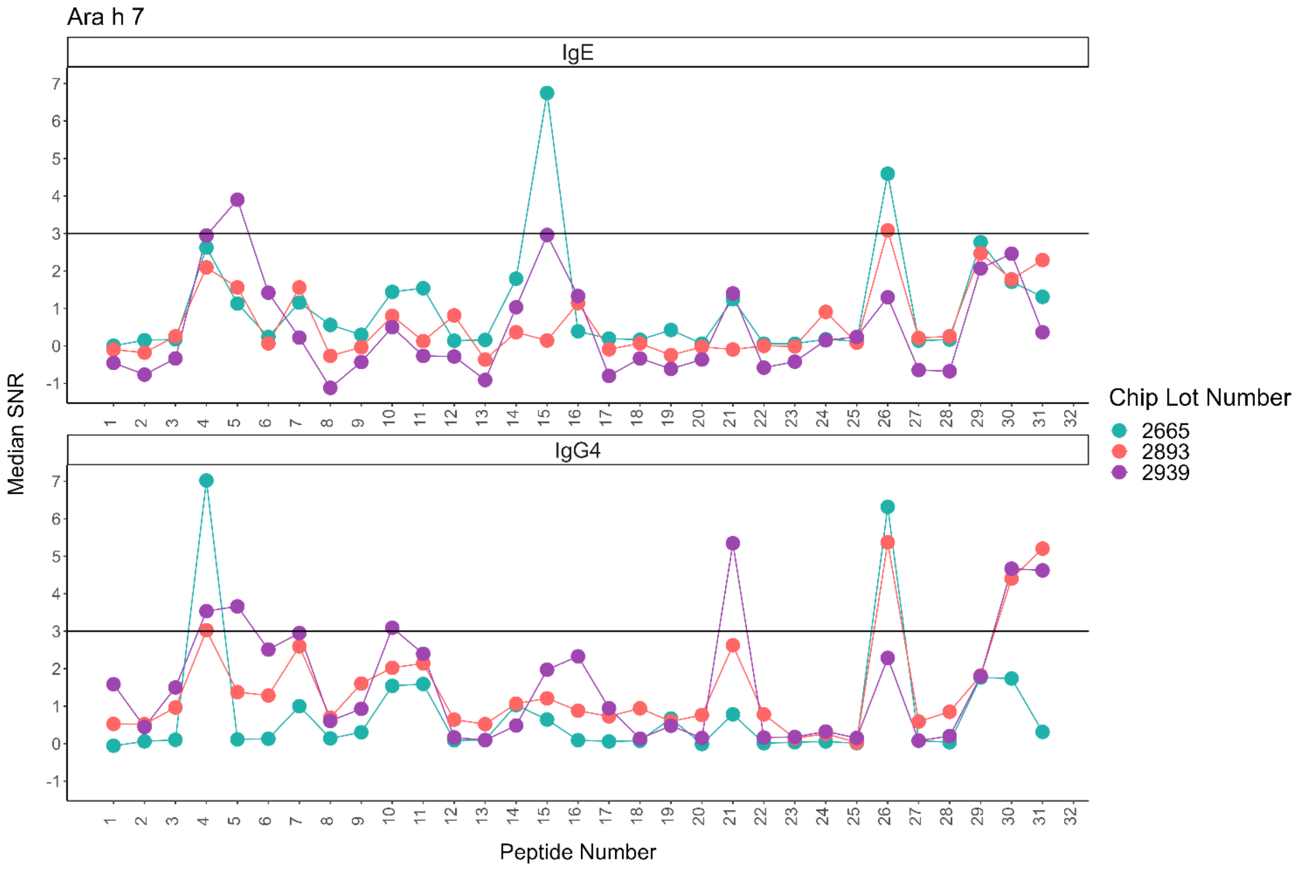


**Supplementary Figure 16. IgE and IgG4 signal to noise (SNR) ratios for Ara h 7, phase 3 pOIT participants.** Major epitopes are indicated by peptides with median SNR > 3 for >50% of participants.

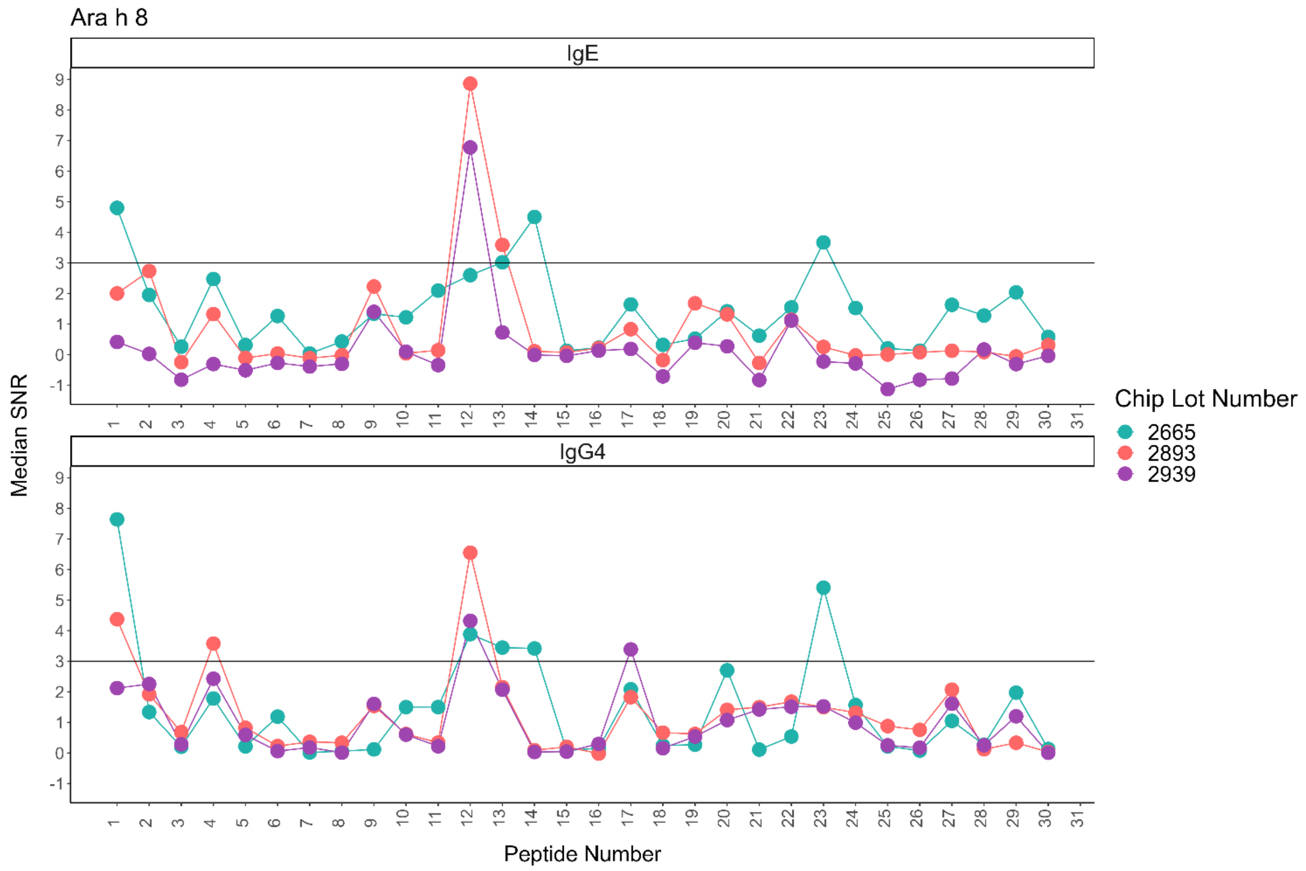


**Supplementary Figure 17. IgE and IgG4 signal to noise (SNR) ratios for Ara h 8, phase 3 pOIT participants.** Major epitopes are indicated by peptides with median SNR > 3 for >50% of participants.

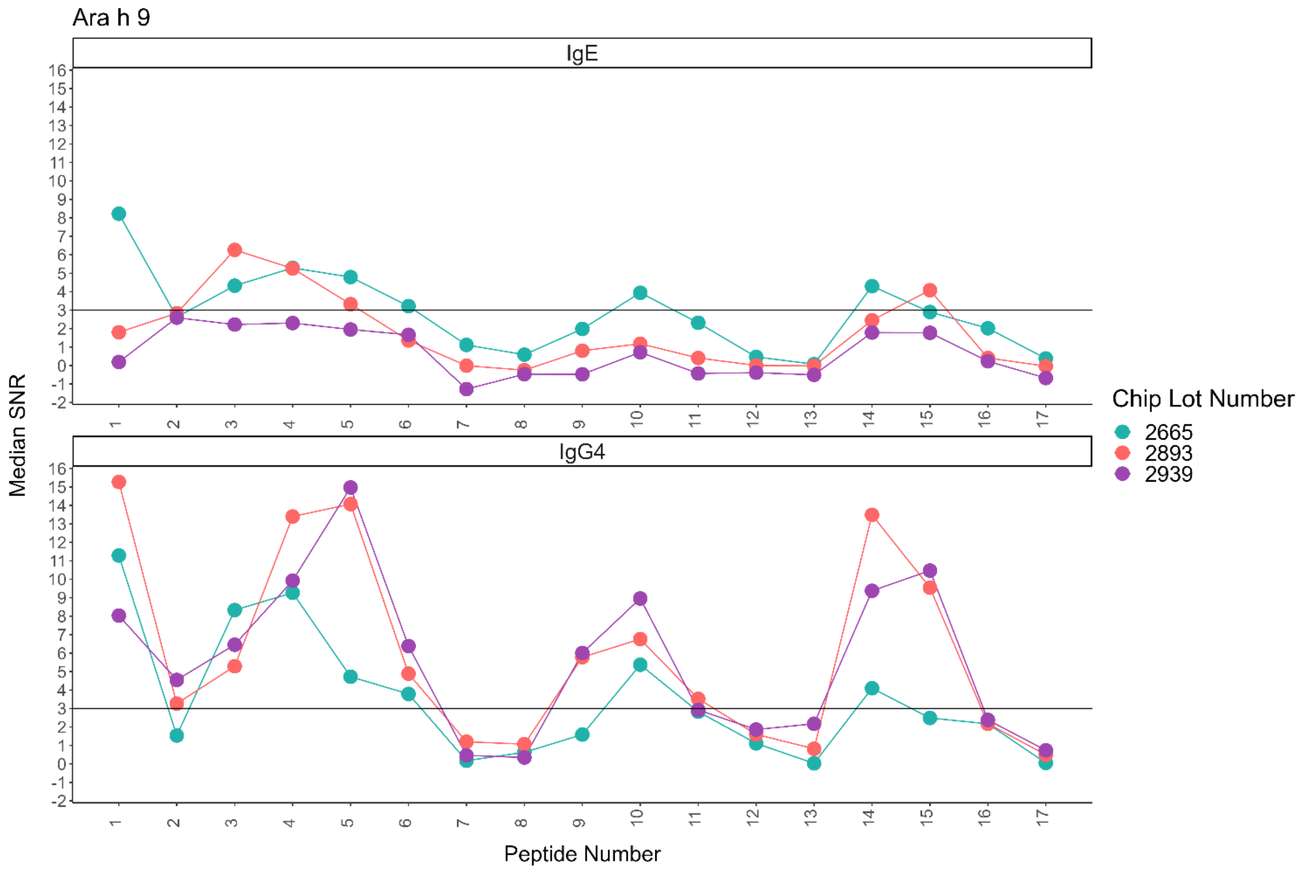


**Supplementary Figure 18. IgE and IgG4 signal to noise (SNR) ratios for Ara h 9, phase 3 pOIT participants.** Major epitopes are indicated by peptides with median SNR > 3 for >50% of participants.

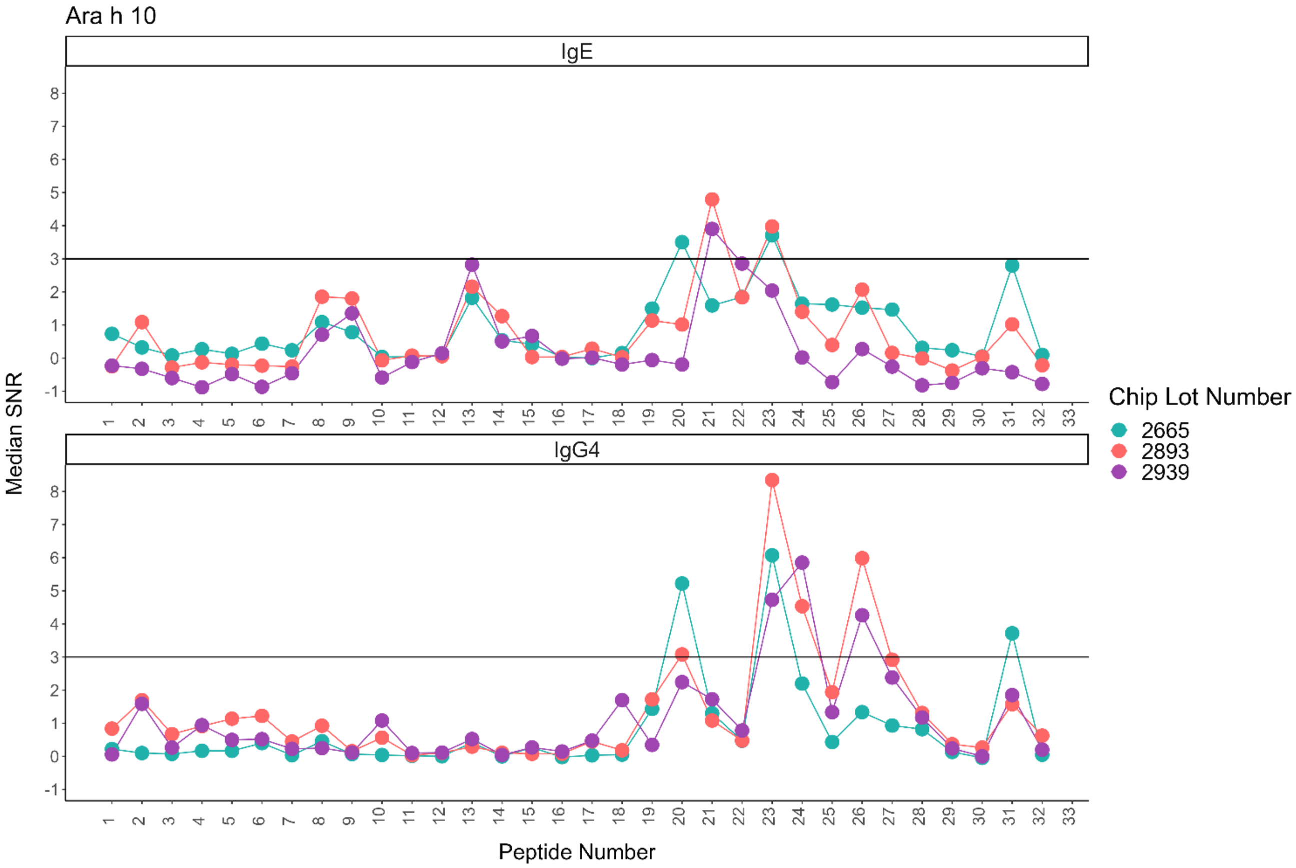


**Supplementary Figure 19. IgE and IgG4 signal to noise (SNR) ratios for Ara h 10, phase 3 pOIT participants.** Major epitopes are indicated by peptides with median SNR > 3 for >50% of participants.

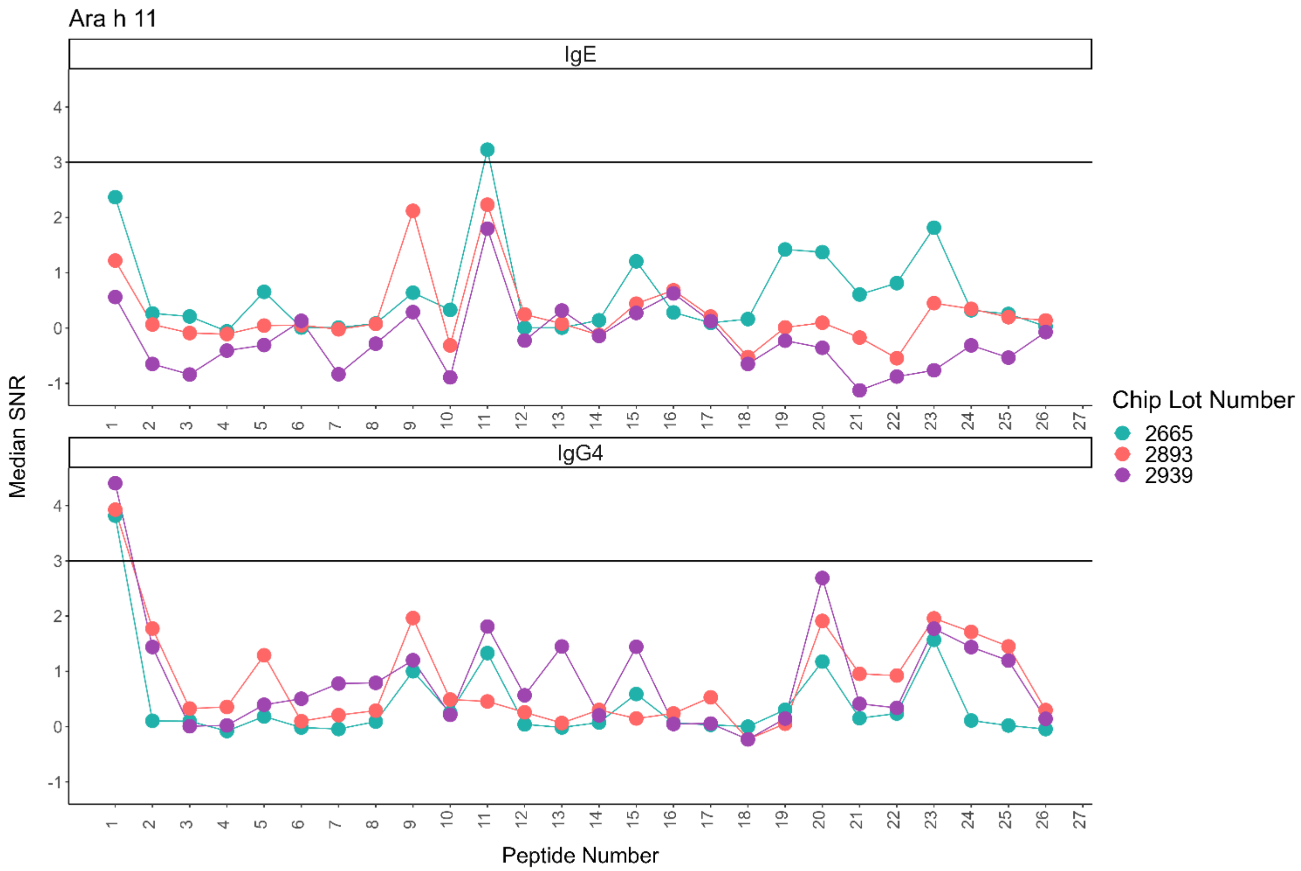


**Supplementary Figure 20. IgE and IgG4 signal to noise (SNR) ratios for Ara h 11, phase 3 pOIT participants.** Major epitopes are indicated by peptides with median SNR > 3 for >50% of participants.

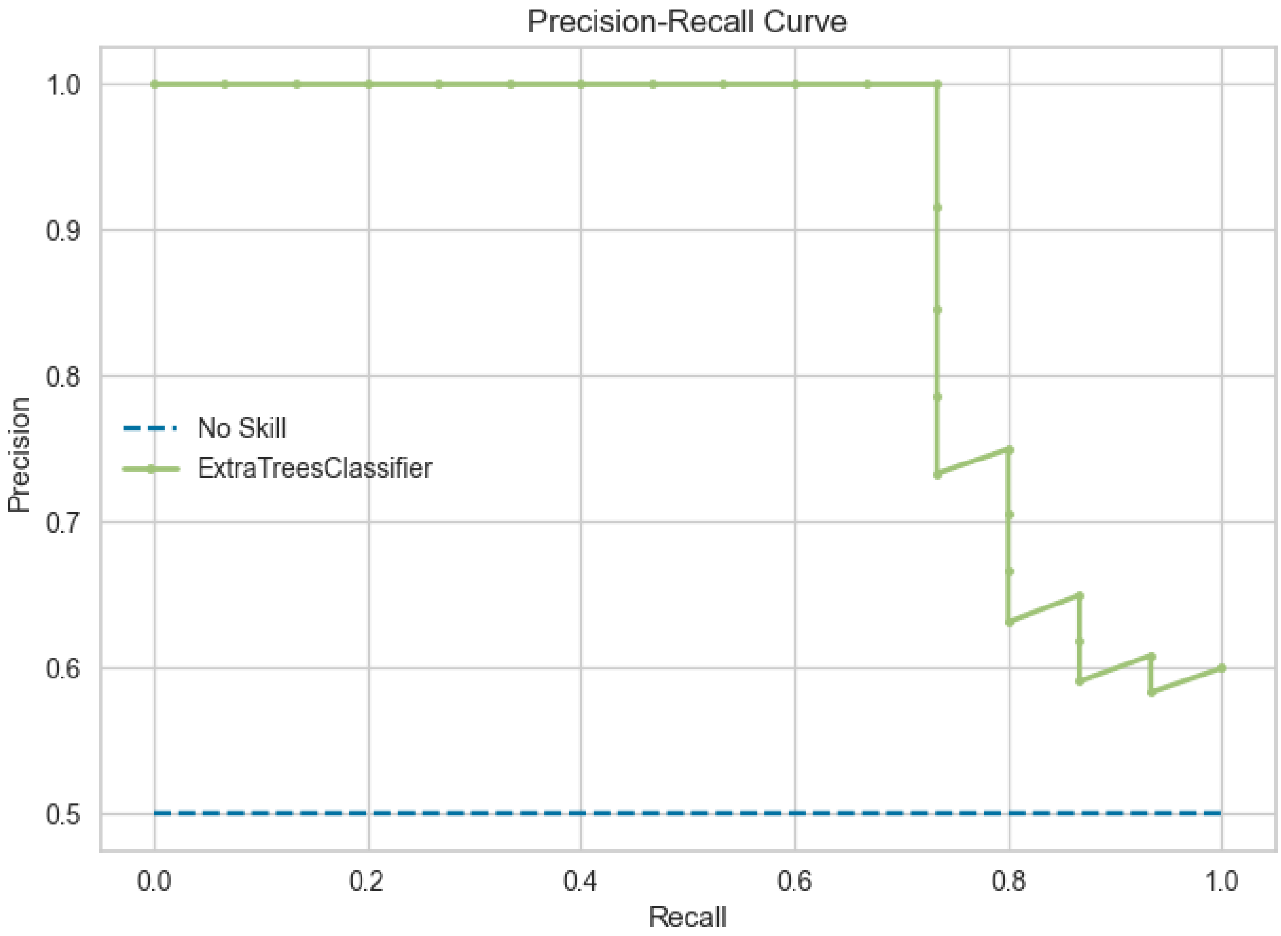


**Supplementary Figure 21. Precision-Recall (PR) curve for finalized Extremely Randomized Trees classifier.** Precision shows how often the model is correct when predicting the target class (timepoint, i.e., desensitization), while recall shows whether the model can find all objects (study participants) of the target class. The dashed line indicates the PR curve of a 'no-skill' classifier that cannot differentiate between different target class labels and predicts random classes or a constant class in all cases.
